# 2D Differential Metallic Immunopotentiators Drive High Diversity and Capability of Antigen‐specific Immunity Against Tumor

**DOI:** 10.1002/advs.202405729

**Published:** 2024-09-03

**Authors:** Hongze Ren, Anqi Zhu, Wei Yang, Yiwen Jia, Hui Cheng, Ye Wu, Zhengqi Tang, Weifan Ye, Mayu Sun, Yujie Xie, Meihua Yu, Yu Chen

**Affiliations:** ^1^ Materdicine Lab School of Life Sciences Shanghai University Shanghai 200444 China; ^2^ School of medicine Shanghai University Shanghai 200444 China; ^3^ Department of Medical Ultrasound Shanghai Tenth People's Hospital School of Medicine Tongji University Shanghai 200070 China; ^4^ Department of Urology Xinhua Hospital School of Medicine Shanghai Jiaotong University Shanghai 200092 China; ^5^ Laboratory Center Shanghai Municipal Hospital of Traditional Chinese Medicine Shanghai University of Traditional Chinese Medicine Shanghai 201203 China

**Keywords:** 2D nanosheets, antigen‐specific immunity, cancer immunotherapy, cancer vaccines, material adjuvants

## Abstract

The therapeutic efficacy of vaccines for treating cancers in clinics remains limited. Here, a rationally designed cancer vaccine by placing immunogenically differential and clinically approved aluminum (Al) or manganese (Mn) in a 2D nanosheet (NS) architecture together with antigens is reported. Structurally optimal NS with a high molar ratio of Mn to Al (MANS‐H) features distinctive immune modulation, markedly promoting the influx of heterogeneous innate immune cells at the injection site. Stimulation of multiple subsets of dendritic cells (DCs) significantly increases the levels, subtypes, and functionalities of antigen‐specific T cells. MANS‐H demonstrates even greater effectiveness in the production of antigen‐specific antibodies than the commercial adjuvant (Alhydrogel) by priming T helper (Th)2 cells rather than T follicular helper (Tfh) cells. Beyond humoral immunity, MANS‐H evokes high frequencies of antigen‐specific Th1 and CD8^+^ cell immunity, which are comparable with Quil‐A that is widely used in veterinary vaccines. Immunized mice with MANS‐H adjuvanted vaccines exert strong potency in tumor regression by promoting effector T cells infiltrating at tumor and overcoming tumor resistance in multiple highly aggressive tumor models. The engineered immunogen with an intriguing NS architecture and safe immunopotentiators offers the next clinical advance in cancer immunotherapy.

## Graphical abstract

Cancer vaccines offer unprecedented advancements in cancer immunotherapy. Here, the impact of the heterogenicity of immunogenically differential aluminum or manganese presented in a 2D nanosheet architecture on the diversity and effectiveness of anti‐tumor immunity is reported. The optimal bimetallic adjuvant exhibits superior anti‐tumor performance than commercial adjuvants in aggressive melanoma and cervical cancer models, enhancing its appeal for clinical use.

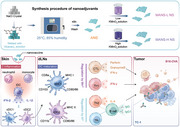

## 1 Introduction

Breakthrough development of new vaccines could save millions of lives, of which adjuvants are essential ingredients in vaccine formulations, harnessing the host immune response against antigens with remarkably enhanced magnitude, longevity, and effectiveness.^[^ ^1^ ^]^ Despite over 100 years old history of the use of adjuvants (e.g., aluminum salts first used in 1926) in human vaccines, only a few adjuvants have been approved in licensed vaccines in clinics to date.^[^ ^2^ ^]^ Numerous innovative adjuvant platforms containing distinct immunostimulants are undergoing explosive exploration to complement the classical adjuvants that hardly trigger an appropriate immune response against a broad spectrum of diseases that are problematic to be prevented or cured, such as cancer, HIV (human immunodeficiency virus), and malaria.^[^ ^1^, ^3^ ^]^ The understanding of the mechanism of action of adjuvants is of great interest, forming the foundation for advances in adjuvant development and vaccinology. Aluminum‐containing adjuvants are known to produce powerful antibody responses and T helper 2 (Th2) cell responses, while having limited cellular immunity. Nonetheless, cellular immune responses, particularly CD8^+^ T cells, are key mediators for the elimination of virus‐infected cells or mutated cancerous cells. To understand how the immune system senses and responds to aluminum‐containing adjuvants, several prevailing mechanisms have been proposed but contested. “Depot theory” proposed by Glenny and co‐workers in 1931 suggested aluminum salts mediated the sustained antigen release at the injection site, prolonging the stimulation of the immune system.^[^ ^4^ ^]^ Recent work highlights the crucial role of innate immune cells in initiating adaptive immune response, whereby the “danger hypothesis” was proposed by Polly Matzinger in 1994. It suggested that cell death from aluminum salt particulate‐caused tissue damage led to the release of danger signals, ultimately evoking the activation of innate immune cells and lymphocytes.^[^ ^5^ ^]^ More recently, inflammasome activation triggered by the phagocytosis of particulate aluminum salts was observed by Veit Hornung and colleagues, which regulated its immunogenicity.^[^ ^6^ ^]^ Huge efforts are still required to fully understand the mechanism of aluminum‐containing adjuvants at cellular and molecular levels.

Another intensively explored chemical element in immune modulation is manganese.^[^ ^7^ ^]^ The pioneering work by Jiang and co‐workers first discovered that manganese ions were indispensable for the host sensing cytosolic double stranded DNA (dsDNA) via cyclic guanosine monophosphate‐adenosine monophosphate synthase (cGAS) and triggering the activation of downstream stimulator of interferon genes (STING) signaling against virus infection.^[^ ^7^ ^]^ Strikingly, manganese ions were found potent immunostimulants by directly activating STING‐type I Interferons (IFNs) pathway in a cGAS‐independent manner.^[^ ^8^ ^]^ The adjuvant activity of manganese ions varies based on their physical forms. It was found that the colloidal form of manganese salts, rather than soluble free ions, effectively stimulates the NLR family pyrin domain containing 3 (NLRP3)‐apoptosis‐associated speck‐like protein containing a C‐terminal caspase recruitment domain (ASC) inflammation pathway. This activation enhances the adjuvant's potency, leading to a strong cellular and humoral immune response, in conjunction with manganese‐STING signaling.^[^ ^9^ ^]^ These findings are of remarkable significance in supporting the clinical studies of manganese salts in cancer immunotherapy,^[^ ^8^ ^]^ igniting enormous research interest in the development of advanced manganese‐based adjuvants.^[^ ^10^ ^]^ Beyond inorganic adjuvants, recent advances in small molecular adjuvants that target other pattern‐recognition receptors (PRRs), such as toll‐like receptor (TLR)7/8 agonist,^[^ ^11^ ^]^ STING agonists of cyclic dinucleotide (CDN)^[^ ^10^, ^12^ ^]^ and MSA‐2,^[^ ^13^ ^]^ have revealed the importance of the formulation of these immunostimulants in micro‐ or nano‐particulates, which dramatically augmented the immunogenicity, reduced the immunological toxicity, and altered their pharmaceutical biodistribution in tissue‐specific innate immune cells (e.g., draining lymph node (dLN) resident DCs^[^ ^14^ ^]^ and tumor‐infiltrating monocytes^[^ ^15^ ^]^). Thin‐layered 2D nanosheet (NS) formulation of immunostimulants is technologically intriguing with unprecedented advancements in cancer immunotherapy^[^ ^16^ ^]^ by offering a high surface area to volume ratio and enhanced nano‐immune interactions. However, it remains unexplored how the heterogenicity of immunogenically differential aluminum or manganese placed in an NS architecture impacts the coordination of innate and adaptive immunity in cancer vaccination.

**Figure 1:**
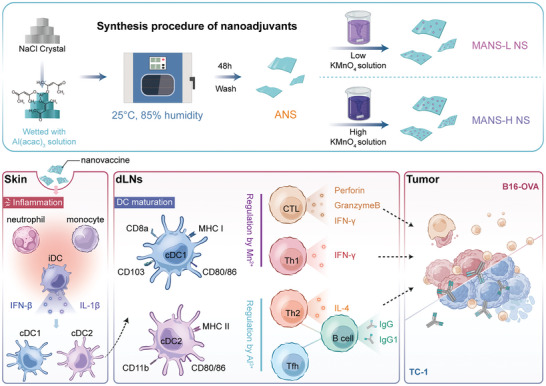
Schematic illustration of the preparation procedures of NSadjuvant with immunogenically differing Mn and Al and action mechanisms by which NS determines the modulation of innate immune cells and the subtypes of antigen‐specific immunity for enhanced anti‐tumor efficacy.

Here, we reported the construction of 2D NS adjuvants with different molar ratios of aluminum to manganese (**Figure** **1** top panel) and explored their adjuvant activity and mechanism of action in cancer vaccines. The results revealed that the bimetallic NS adjuvant with a high ratio of manganese to aluminum (MANS‐H) markedly triggered the recruitment of heterogenous subsets of innate immune cells at skin post‐injection, such as proinflammatory macrophages, monocytes, and neutrophils (Figure 1 bottom panel). MANS‐H exhibited superior performance in enhancing antigen uptake and activating distinct subsets of DCs, including type 1 conventional DCs (cDC1, CD103^+^ migratory DCs, and CD8^+^ resident DCs), type 2 conventional DCs (cDC2, CD11b^+^ DCs) and Langerhans cells (LCs) via stimulation of STING‐type I IFN and Interleukin‐1 beta (IL‐1β) inflammation pathways. Mechanically, Mn showed remarkably greater effectiveness in priming antigen‐specific IL‐4^+^CD4^+^ Th2, IFN‐γ^+^CD4^+^ Th1, and multifunctional CD8^+^ T cells, which further developed into memory phenotypes. By contrast, Al showed potency in evoking the production of CXCR5^+^CD4^+^ Tfh and antibodies. Mice vaccinated with MANS‐H adjuvanted vaccines demonstrated significant tumor suppression by promoting the frequency of tumor‐infiltrating CD8^+^ cells with distinct activated phenotypes and cytotoxic natural killer (NK) cells while suppressing the suppressive innate immune cells and CD4^+^Foxp3^+^ Tregs at the tumor site. The potent therapeutic efficacy of MANS‐H combined with tumor‐associated or specific antigens was validated in aggressive murine cancer models, indicating the great potential of bimetallic oxides NS adjuvant cancer vaccine immunotherapy for enhancing clinical efficacy.

## 2 Results and Discussion

### 2.1 Preparation and Characterization of Several Nanosheet Adjuvants

**Figure 2:**
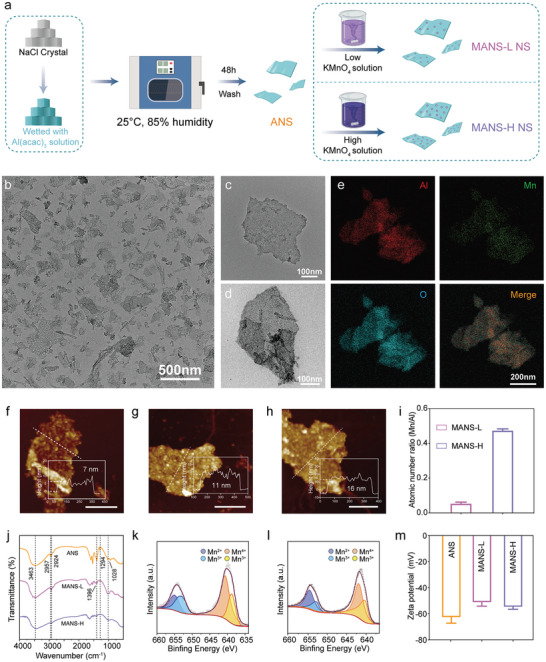
Characterization of nanosheet adjuvants. a) Schematic illustration of the preparation of ANS, MASN‐L, and MANS‐H. b–d) TEM images of b) ANS, c) MANS‐L, and d) MANS‐H. e) The element mapping images of MANS‐H. f–h) AFM topography images and height profiles of f) ANS, g) MANS‐L, and h) MANS‐H. Scar bar: 200 nm. i) The atomic ratios of Mn/Al in MANS‐L and MANS‐H were determined by ICP analysis (*n =* 3). j) FTIR spectra of ANS, MANS‐L, and MANS‐H. k‐l) XPS spectra of k) MANS‐L and l) MANS‐H. m) Zeta potential values of ANS, MANS‐L, and MANS‐H in PBS solution (*n =* 3). Data are presented as mean ± SD (i and m).

MANS adjuvants were prepared via a facile hard template method as illustrated in **Figure** **2**a. Briefly, NaCl microcrystals (Figure S1, Supporting Information) were wetted with aluminum acetylacetonate toluene solution, which underwent hydrolytic polycondensation in a humidity chamber to form aluminum containing NS (ANS) after washing with water. ANS directly reacted with potassium permanganate (KMnO_4_) aqueous solution to yield MANS with low or high manganese to aluminum ratios (denoted as MANS‐L and MANS‐H). Transmission electron microscopy (TEM) images revealed a thin layered structure of ANS with a size ≈3–4 µm and a smooth surface (Figure S2a, Supporting Information). Consistently, scanning electron microscope (SEM) images of ANS displayed micrometer‐sized layers (Figure S2b, Supporting Information). Following ultrasonication, ANS was downsized to 200–500 nm (Figure 2b; Figure S2, Supporting Information). After incorporating Mn, MANS‐L (Figure 2c) and MANS‐H (Figure 2d) maintained a nanosheet structure, and the elemental mapping images of MANS‐H (Figure 2e) implied that Mn was uniformly doped in ANS. Atomic force microscopy (AFM) image further confirmed the lamellar structure of ANS with a thin thickness of 7 nm (Figure 2f), the thickness of MANS‐L increased to 11 nm (Figure 2g) and MANS‐H increased to 16 nm (Figure 2h), contributed by the doping layer of Mn. Inductively coupled plasma emission spectrometer (ICP) analysis (Figure 2i) showed that the quantified molar ratios of Mn to Al were 0.05 ± 0.01 in MANS‐L and 0.46 ± 0.01 in MANS‐H.

Fourier transform infrared (FTIR) spectroscopy analysis was performed to characterize the chemical composition of ANS, MANS‐L, and MANS‐H. FTIR spectrum of ANS (Figure 2j) exhibited characteristic absorption peaks at 1294 cm^−1^ (C–O stretching), 1396 cm^−1^(O–H bending), 2924 cm^−1^ (C–H stretching), and 2957 cm^−1^ (C–H stretching), suggesting partially unhydrolyzed C‐O, O‐H and C–H groups derived from the Al precursors. The typical peaks at 1028 cm^−1^ (δ_s_(Al‐O‐H)) and 3463 cm^−1^ (ν_as_(Al–O–H)) implied the formation of AlOOH.^[^ ^17^ ^]^ By contrast, the typical peaks of C–O, O–H, and C–H groups in MANS‐L and MANS‐H were less distinguished compared to those in ANS, indicating the enhanced hydrolysis of Al precursor residual during the oxidation‐reduction reaction with KMnO_4_ solution. X‐ray photoelectron spectroscopy (XPS) was used to assess the valence states of Mn in MANS‐L and MANS‐H. The XPS survey of MANS‐L and MANS‐H (Figure S3, Supporting Information) displayed characteristic peaks of Mn and Al with an atomic ratio of 0.14 and 0.81, respectively, which were 2–3 times those values with ICP analysis, implying that Mn is predominately decorated on the surface of ANS. XPS spectra of Mn 2p_3/2_ from both MANS‐L and MANS‐H exhibited characteristic peaks at 654.6 eV for Mn^2+^, 653.0 eV and 641.5 eV for Mn^3+^, and 643.0 eV for Mn^4+^ (Figure 2k,l), suggesting mixed valence states of Mn. Additionally, the zeta potential of three NS adjuvants was negative in phosphate buffered solution (PBS) (Figure 2m). Collectively, several NS adjuvants with varied Mn/Al ratios were successfully fabricated.

### 2.2 MANS‐H Adjuvant Promotes the Maturation of Antigen‐presenting Cells (APCs) via Activating cGAS‐STING and Inflammasome Signaling Pathway

**Figure 3:**
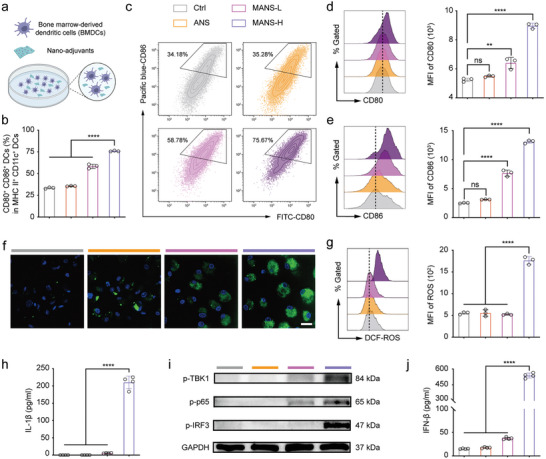
MANS‐H adjuvant potentially triggers BMDC maturation, ROS production, and activation of the inflammasome and STING‐IFN‐β pathway. a) Illustration shows the procedure to induce the BMDC maturation (created with permission by BioRender). b) Flow cytometry shows the proportion of CD80^+^ CD86^+^ DCs (in the MHC II^+^ CD11c^+^ DCs) treated with different NS adjuvants (*n =* 3). c) Representative counterplot of CD80^+^ CD86^+^ DCs. d‐e) Representative histogram overlays and median fluorescence intensity (MFI) of the expression of d) CD80 and e) CD86 on BMDCs after incubation with ANS, MANS‐L or MANS‐H (*n =* 3). f) Confocal microscopy images and g) flow cytometry analysis shows intracellular ROS levels in BMDCs induced by ANS, MANS‐L, or MANS‐H (*n =* 3). h) ELISA analysis summarizes IL‐1β secretion levels in BMDMs treated with PBS or NS adjuvants for 48 h (*n =* 4). i) Western blot analysis shows the expression levels of pTBK1, p‐p65 and ‐IRF3 in BMDCs treated with PBS or NS adjuvants for 20 h (n = 3). j)ELISA analysis of IFN‐β secretion levels in the supernatants of BMDCs (*n =* 4). Data are presented as means ± SD. P values were determined by a one‐way ANOVA test. ns, *p >* 0.05, **p < 0.01, ****p < 0.0001.

DCs are a crucial subset of APCs, which are speciliazed in antigen capture and presentation to prime adaptive immune cells.^[^ ^18^ ^]^ Bone marrow‐derived DCs (BMDCs) were co‐cultured with ANS, MANS‐L, or MANS‐H to assess their adjuvant activities (**Figure** **3**a). MANS robustly promotes the proportion of CD80^+^ CD86^+^ DCs (Figure 3b,c). The flow cytometry results displayed that ANS slightly upregulated the expression of co‐stimulatory markers of CD80 and CD86. By contrast, BMDCs co‐cultured with MANS‐L or MANS‐H expressed a significantly higher level of CD80 (Figure 3d) and CD86 (Figure 3e), compared with control and ANS groups, particularly MANS‐H. These results suggest that the maturation of BMDCs profoundly depends on the dose of Mn contained in the NS adjuvants. Previous reports showed that Al‐containing rods were able to effectively activate BMDCs into a mature state under a greatly higher concentration (100 versus 15 µg mL^−1^).^[^ ^17^ ^]^ Regarding BMDC stimulation, Mn exhibited superior adjuvanticity over Al.

Reactive oxygen species (ROS) are key stimulants triggering the activation of inflammation signals and the release of pro‐inflammatory cytokines, which are essential in adjuvant activity.^[^ ^19^ ^]^ To explore the cellular mechanism of MANS‐H adjuvant effect, we next examined whether metal ions were able to induce intracellular ROS. BMDCs were treated with ANS, MANS‐L, or MANS‐H and then co‐cultured with ROS probe dichlorofluorescin diacetate (DCF‐DA). The confocal microscopy images showed that MANS‐H but not ANS or MANS‐L exhibited strong green fluorescence signals, implying that MANS‐H markedly promoted the production of intracellular ROS (Figure 3f). The quantified results from flow cytometry (Figure 3g) confirmed a consistent trend in ROS generation, which was mainly contributed by the Mn‐Fenton reaction.^[^ ^20^ ^]^ We next performed western blot (WB) and enzyme‐linked immunosorbent assay (ELISA) analysis to test whether ROS mediates the activation of IL‐1β inflammation signal pathway in BM‐derived macrophages (BMDMs) that are appropriate in inflammation assessment.^[^ ^9^, ^21^ ^]^ The results demonstrated that MANS‐H but not ANS or MANS‐L significantly promoted the secretion of proinflammatory cytokine IL‐1β (Figure 3h) in lipopolysaccharide (LPS)‐primed BMDMs, suggesting that MANS‐H potently activated inflammasome‐IL‐1β signaling pathway.

The activity of STING agonist Mn^2+^ was reported to be dependent on its dose and physical forms,^[^ ^9^ ^]^ thus, we explored the capability of NS containing Al/Mn in STING activation. MANS‐H but not ANS significantly promoted downstream signaling of STING pathway by apparently increasing the expression of phosphorylated tank‐binding kinase 1 (TBK1), interferon regulatory factor 3 (IRF3), and p65 (Figure 3i; Figure S4, Supporting Information), thereby significantly stimulating the production of type I IFN‐β in the supernatants (Figure 3j). Of note, MANS‐L moderately upregulated the expression of STING downstream signaling molecules, which inadequately triggered the secretion of IFN‐β (Figure 3i,j). The calculated dose of Mn derived from MANS‐H was 0.037 mm, which was much lower than the effective dose in Mn salts (0.2 mm),^[^ ^9^ ^]^ implying that the superior potency of MANS‐H might be contributed by the NS structure. We previously observed that Mn‐based nanoparticles with a high oxidative status of Mn^4+^ showed pH‐buffering capability in the acidic endo‐lysosomal compartments,^[^ ^21^ ^]^ which might slow down the degradation of STING.^[^ ^22^ ^]^ It therefore could be the reason why MANS‐H showed significantly enhanced downstream activity of STING activation and IFN‐β secretion than soluble Mn^2+^ ions. In addition, Mn^2+^ released from the nanosheet structure provided local concentrated STING agonists, which potentially led to multivalent interactions with STING.

**Figure 4:**
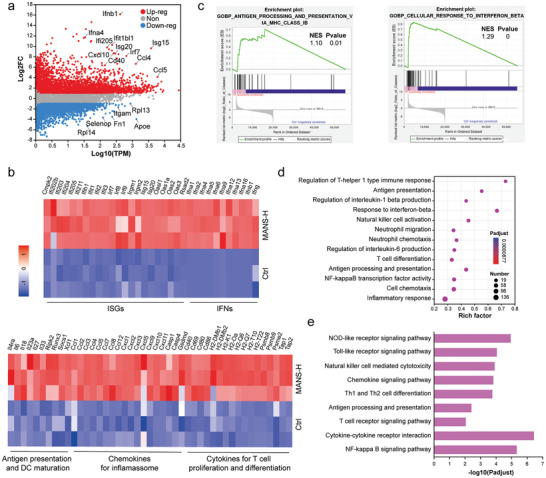
Bulk RNA sequencing analysis of BMDCs treated with PBS or MANS‐H. a) Volcano plot shows the differentially expressed genes that are significantly or non‐significantly upregulated or downregulated in BMDCs treated with MANS‐H by comparing with the PBS group. b). Heatmaps summarize the selected genes related to the STING‐IFN‐β pathway (ISGs and IFNs), cytokines for T cell proliferation and differentiation, chemokines for inflammasome, antigen presentation, and DC maturation. c) Gene set enrichment analysis (GSEA), d) KEGG, and e) GO enrichment analysis of differentially expressed genes associated with innate and adaptive immune response.

To further investigate the immune modulation mechanism of MANS‐H, we used transcriptomics RNA sequencing to analyze BMDCs treated with MANS‐H. A total 23 899 genes were investigated, of which 3222 genes were significantly upregulated in BMDCs treated with MANS‐H compared to the control group (Figure S5, Supporting Information). The top upregulated genes mainly include interferon‐stimulated genes (Isg15, Isg20, and Ifi205) and chemokines (Ccl4 and Ccl5), which strongly suggested the stimulation of IFN and inflammation signals (**Figure** **4**a). Interestingly, genes related to M2 anti‐inflammatory phenotypic macrophages (Apoe and Fn1) were observed to be remarkably downregulated (Figure 4a). The representative significantly upregulated genes associated with STING downstream signals of ISGs and IFNs were visualized in heatmaps (Figure 4b), which were consistent with the WB analysis. In addition, sets of significantly upregulated genes were found related to antigen presentation (H2‐Q6, H2‐Q7, and Tap1/2 involved in antigen‐MHC class I complexes) and DC maturation, chemokines (Ccl2/3 and Cxcl5/9) for inflammasome, and cytokines (Il6, Il33 and Il18) for T cell proliferation and differentiation. We found that top‐regulated genes were significantly enriched in cellular response to interferon beta and antigen processing and presentation via major histocompatibility complex (MHC) class I pathways (Figure 4c). Gene ontology (GO) enrichment analysis and Kyoto encyclopedia of genes and genomes (KEGG) pathway enrichment analysis were performed. Compared to the control group, immune‐associated signaling pathways were found prominent in the chemokine signaling pathway, Th1 and Th2 cell differentiation and regulation of interleukin‐1 beta production and cytokine‐cytokine receptor interaction, contributing to the potent adjuvant functions of MANS‐H (Figure 4d,e; Figure S6, Supporting Information).

### 2.3 Differential Immunogenicity of Distinct NS Adjuvants in Recruitment of Heterogenous Subsets of Innate Immune Cells at Injection Site and Activation of Distinct Subsets of DCs In vivo

**Figure 5:**
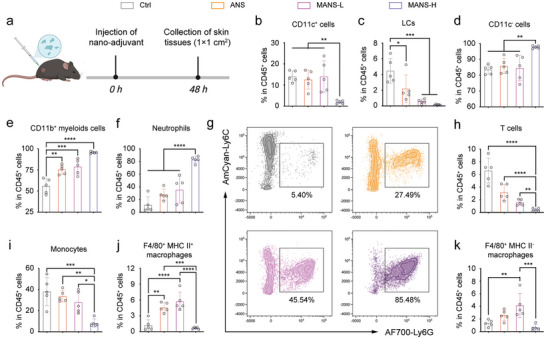
MANS‐H adjuvant dramatically promotes the infiltration of heterogeneous subsets of innate immune cells at the injection site. a) The experimental timeline: C57BL/6J mice subcutaneously received 300 µg of ANS, MANS‐L or MANS‐H or PBS (Ctrl) at the injection site and skin tissues (1 cm^2^) were harvested at 48 h for flow cytometry analysis (created with permission by BioRender). b‐k) The percentages of b) CD11c^+^ cells, c) LCs, d) CD11c^−^ cells, e) CD11b^+^ myeloids, f) neutrophils, h) T cells, i) monocytes, j) F4/80^+^ MHC II^+^ macrophages, k) F4/80^+^ MHC II^−^ macrophages (*n =* 4). g) Representative dot plot of neutrophils in the injection site with different treatment. Data are presented as means ± SD. P values were determined by a one‐way ANOVA test. ns, *p >* 0.05, *p < 0.05, **p < 0.01, ***p < 0.001, ****p < 0.0001.

Adjuvant function in large measure by creating the local pro‐inflammatory environment and triggering the activation of DCs, which is a prerequisite for initiating antigen‐specific adaptive immune responses.^[^ ^23^ ^]^ To compare the adjuvant activity of NSs with immunogenically different ratios of Mn to Al elements in vivo, immunocompetent C57BL/6J mice were subcutaneously injected with ANS, MANS‐L, and MANS‐H, and then flow cytometry analysis was performed to characterize the cellular influx at the injection site (**Figure** **5**a). At 48 h post‐injection, immunization of MANS‐H but not ANS or MANS‐L resulted in a significant decrease in the frequency of CD11c^+^ DCs in the skin (Figure 5b; Figure S7a, Supporting Information). In line with the observation in CD11c^+^ DCs, EpCAM^+^MHC II^+^ Langerhans cells (LCs) dramatically declined in all NS‐vaccinated skin (Figure 5c), indicating the migration of DCs and LCs into dLNs following activation. Differently, we observed a dramatic increase in CD11c^−^ non‐DC population in MANS‐H but not ANS or MANS‐L immunized skin (Figure 5d). Further analysis was performed on the subsets of CD11c^−^ non‐DCs, which revealed that CD11b^+^ myeloid cells predominately contributed to the substantial increase in cellular influx in all NS‐vaccinated skin, particularly MANS‐H (Figure 5e). Additionally, MANS‐H markedly promoted the infiltration of proinflammatory CD11b^+^Ly6G^+^ neutrophils (Figure 5f,g), which was likely mediated by MANS‐Hinduced ROS.^[^ ^24^ ^]^ However, skin T cells markedly decreased (Figure 5h), implying the motility of T cells within the lymphatic capillaries in response to NS‐induced inflammation.^[^ ^25^ ^]^ A substantial decline was also observed in the percentage of CD11b^+^Ly6G^−^ monocytes (Figure 5i) in the skin immunized with MANS‐H, which was possibly caused by their differentiation into skin‐specific macrophages or DCs.^[^ ^26^ ^]^ Interestingly, the immunization of ANS or MANS‐L but not MANS‐H enhanced the influx of the sub‐population of CD11b^+^Ly6G^−^Ly6C^−^ myeloid‐derived cells (Figure S7b, Supporting Information), the function of which has been rarely explored with a possibility of acting as an immune suppressor.^[^ ^27^ ^]^ A similar trend was observed in macrophages comprising distinct subsets of F4/80^+^MHC II^+^ macrophages (Figure 5j) and F4/80^+^MHC II^−^ macrophages (Figure 5k).

**Figure 6:**
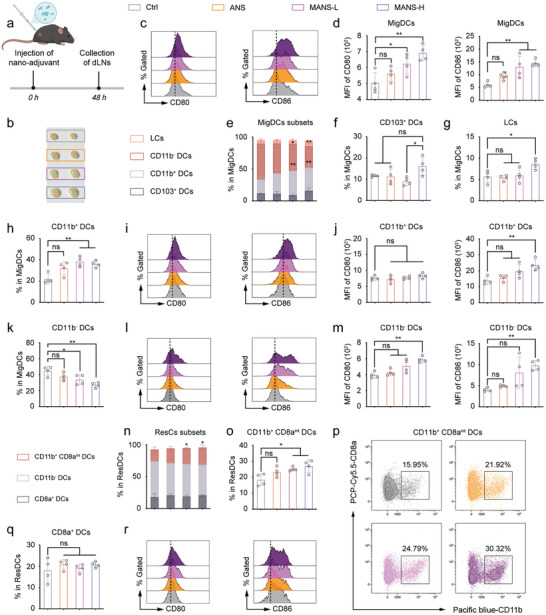
MANS‐H adjuvant remarkably triggers the maturation of distinct phenotypes of MigDCs and ResDCs. a) The experimental timeline: C57BL/6J mice subcutaneously received 300 µg of ANS, MANS‐L or MANS‐H, or PBS (Ctrl), and dLNs were harvested at 48 h for flow cytometry analysis (created with permission by BioRender). b) Representative images of inguinal LNs post different treatments. c,d) Representative histogram overlays c) and MFI of CD80 and CD86 d) on MigDCs derived from inguinal LNs (*n =* 4). e) The proportion sum of MigDC subsets and the percentages of each subset. f–n) Flow cytometry analysis shows the percentage of CD103^+^ DCs f), LCs g), CD11b^+^ DCs h) and CD11b^−^ DCs k), histogram plots of CD80 and CD86 of CD11b^+^ DCs i) and CD11b^−^ DCs l) and MFI of CD80 and CD86 of CD11b^+^ DCs j) and CD11b^−^ DCs m) (*n =* 4). n) The proportion sum of ResDC subsets and the percentages of each subset. o‐r) Flow cytometry analysis shows the percentage of o) CD11b^+^CD8a^int^ DCs, q) CD8a^+^ DCs, representative dot plots of p) CD11b^+^CD8a^int^ DCs, MFI of CD80 and CD86 of r) CD11b^+^CD8a^int^ DCs (*n =* 4). Data are presented as means ± SD. P values were determined by a one‐way ANOVA test. ns, *p >* 0.05, *p < 0.05, **p < 0.01.

DCs comprise highly heterogenous subgroups, of which CD11b^+^ cDC2 and CD103^+^ (CD8a^+^) cDC1s are exceedingly specialized in MHC class II and MHC class I antigen presentation for priming CD4^+^ T cells and CD8^+^ T cells, respectively.^[^ ^28^ ^]^ The discriminated functions of DCs determine or shape the subtypes of adaptive immunity in vaccination.^[^ ^29^ ^]^ The effectiveness of T cell priming heavily relies on the level of DC maturation, such as the upregulation of co‐stimulatory molecules and secretion of cytokines that support T cell proliferation and differentiation.^[^ ^30^ ^]^ To understand the influence of ANS, MANS‐L, and MANS‐H on DCs, we analyzed the phenotypes of DCs in skin dLNs following immunization (**Figure** **6**a). The results demonstrated that MANS‐H and MANS‐L but not ANS immunized groups displayed an obvious increase in dLN size (Figure 6b), which was likely caused by the rapid recruitment of APCs and other sub‐populations of innate immune cells from the skin, such as CD11c^+^ DCs and LCs (Figure 5b,c). Flow cytometry analysis on the cellularity of skin dLNs showed that vaccination with MANS‐H led to a significant decrease in the frequency of migratory DCs (MigDCs, MHC II^high^CD11c^+^, Figures S8 and S9a, Supporting Information).^[^ ^31^ ^]^ MigDCs exhibited marked upregulation of CD80 and CD86 molecules, suggesting potent DC activation. In comparison with MANS‐H, MANS‐L showed a similar level of MigDC maturation, but ANS induced semi‐maturation of MigDCs with promoted expression of co‐stimulatory markers that were statistically non‐significant (Figure 6c,d). The trend of distinct NSs in DC maturation was consistent with the observations in BMDCs. In further phenotyping analysis, MigDCs were subdivided into subsets of LCs, CD11b^+^ cDCs (cDC2), CD11b^−^ DCs and migratory CD103^+^ DCs (cDC1) (Figure 6e). Importantly, MANS‐H but not ANS or MANS‐L significantly promoted the frequencies of CD103^+^DCs (Figure 6f) and immature LCs (Figure 6g; Figure S9b, Supporting Information). The observation of downregulation of CD80 and CD86 on CD103^+^DCs (Figure S9c, Supporting Information) suggested a tolerogenic phenotype, which might have contributed to the absence of antigen presentation (will be further discussed in the next section). All NS adjuvants significantly enhanced the recruitment and maturation of CD11b^+^DCs (Figure 6h–j). The immunization of MANS‐H and MANS‐L but not ANS resulted in a decrease in the number of mature CD11b^−^ DCs (Figure 6k–m). These results implied that the decrease in MigDCs was mainly caused by a marked decrease of CD11b^−^ DCs. MANS‐H with a high ratio of Mn/Al exhibited superior performance in the effective activation of multiple subsets of MigDCs.

Cooperation between MigDCs and resident DCs (ResDCs, MHC II^int^CD11c^+^) in skin‐dLNs allows optimization of DC capability responding to stimulatory vaccines or infection,^[^ ^29^, ^31^ ^]^ thus we next assessed the impact of NS adjuvants on ResDCs, which were found in an unexpanded and immature state (Figures S8 and S10a, Supporting Information). ResDCs were then subdivided into three phenotypes: CD8a^+^ DCs, CD11b^+^CD8a^int^ DCs, and CD11b^−^ DCs (Figure 6n). MANS‐L and MANS‐H but not ANS induced a significant increase in CD11b^+^CD8a^int^ DCs (Figure 6o,p) rather than the other two subsets (Figure 6q; Figure S10b, Supporting Information). Interestingly, only MANS‐H was efficient to induce a significant upregulation of CD80 and moderate promotion of CD86 (Figure 6r; Figure S10c, Supporting Information) expressed on CD11b^+^CD8a^int^ DCs, but not on CD11b^−^ DCs (Figure S10b, Supporting Information) or CD8a^+^ DCs (Figure S10d, Supporting Information). This effect would potentially contribute to the cross‐presentation capability of MANS‐H adjuvanted vaccines.

Collectively, we answered the question of how the differential immunogenicity of distinct NS adjuvants regulated the maturational states of dLN‐DCs with highly phenotypic and spatial heterogeneity. MANS‐H is the most pronounced adjuvant for adequately stimulating the activation of MigDCs and ResDCs, which contain both cDC1 and cDC2 for broad T‐cell priming.

### 2.4 Enhanced Antigen Delivery to DCs and Antigen Cross Presentation Mediated by Distinct NS Adjuvants In vitro and In vivo

**Figure 7:**
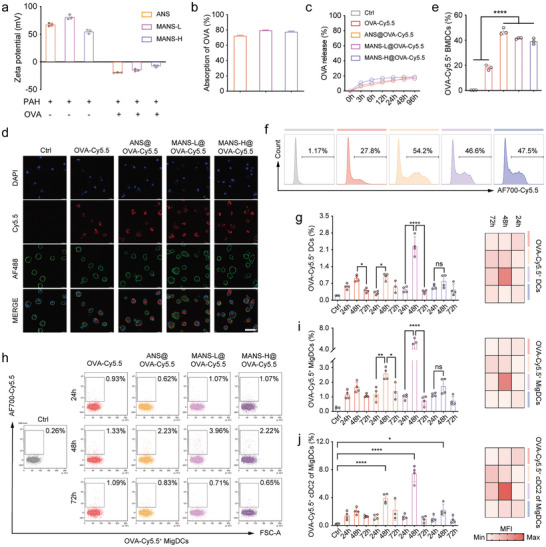
Dynamic cellular biodistribution of NS adjuvanted OVA‐Cy5.5 vaccines. a) Zeta potential values of three NS adjuvants before and after forming complexes with PAH or OVA (*n =* 3). b) OVA absorption efficiency and c) release profiles of three NS adjuvants (*n =* 3). d) Representative confocal microscopy images of BMDCs after incubation with NS@OVA‐Cy5.5 (red) and staining with DAPI (nucleus, blue) and iFluorTM 488 phalloidin (F‐actin in membrane, green). Scar bar: 40 µm. e) Bar charts and f) representative histograms from flow cytometry analysis show the proportions of OVA‐Cy5.5^+^ BMDCs after incubation with NS@OVA‐Cy5.5 for 6 h (*n =* 3). g‐j) Flow cytometry analysis shows the proportions of g) OVA‐Cy5.5^+^DCs, i) OVA‐Cy5.5^+^MigDCs, and j) OVA‐Cy5.5^+^CD103^−^DCs in dLN‐DCs, corresponding MFI heatmaps, and h) representative dot plots of OVA‐Cy5.5^+^MigDCs at different timepoints (*n =* 3 or 4). Data are presented as means ± SD. P values were determined by a one‐way ANOVA test. ns, *p >* 0.05, *p < 0.05, **p < 0.01, ***p < 0.001, ****p < 0.0001.

To evaluate the antigen delivery capability of ANS, MANS‐L, and MANS‐H, the negatively charged NS adjuvants (≈55 mV) were coated with positive poly(allylamine hydrochloride) (PAH),^[^ ^32^ ^]^ which turned to be positively charged (≈ +60–80 mV, **Figure** **7**a) to promote the adsorption of ovalbumin (OVA, a model antigen) via electrostatic interactions. All NS@OVA samples exhibited a negative surface charge (≈10 mV, Figure 7a), indicating successful loading of OVA. Absorption rates of OVA for all NS exceed 70% (Figure 7b), corresponding to a loading capability of 70 µg OVA per 1 mg NSs (Figure S11a, Supporting Information). To track antigens in vitro and in vivo, OVA was conjugated with Cy5.5 (OVA‐Cy5.5), which displayed a slow antigen release from NSs with only ≈20% release up to 96 h (Figure 7c), implying stable vaccine formulations. It is vital to avoid the induction of self‐tolerance by free antigens.^[^ ^21^ ^]^

To assess intracellular antigen delivery in vitro, BMDCs were co‐cultured with distinct NS@OVA‐Cy5.5 and then analyzed using confocal microscopy and flow cytometry. The confocal microscopy images revealed that OVA‐Cy5.5 was readily delivered into the cytoplasm of BMDCs (Figure 7d). The quantified data by flow cytometry analysis demonstrated that OVA‐Cy5.5^+^ BMDCs in all NS@OVA‐Cy5.5 groups reached ≈50%, which was significantly higher than that of free OVA‐Cy5.5 (Figure 7e,f). However, ANS@OVA‐Cy5.5 displayed a markedly higher Cy5.5 MFI value than MANS‐L@OVA‐Cy5.5 and MANS‐H@OVA‐Cy5.5 (Figure S11b, Supporting Information), indicating enhanced cellular uptake of thinner ANS in vitro.

We next determined the dynamic cellular biodistribution of OVA‐Cy5.5 in skin dLN DCs in C57BL/6J mice (Figure S12, Supporting Information). All OVA‐Cy5.5 groups showed a steady and significant increase in the proportions of OVA‐Cy5.5^+^CD11c^+^MHC II^+^ DCs in skin dLNs from 24 to 48 h post‐injection (Figure 7g left) but a dramatic decline at 72 h. Of note, the frequency of OVA‐Cy5.5^+^ DCs and MFI value (Figure 7g right; Figure S13a, Supporting Information) at 48 h was found prominent in MANS‐L@OVA group, which was probably caused by the combined effect of thin thickness‐mediated cellular uptake and Mn‐facilitated DC maturation and migration. A similar trend was observed in the subsets of MigDCs (Figure 7h,i; Figure S13b, Supporting Information) and ResDCs (Figure S13c, Supporting Information). However, OVA‐Cy5.5 positive populations in MigDCs were round 2–3 times those in ResDCs across experimental time, suggesting that MigDCs were the primary contributor to sampling and presenting antigens. In addition, the observed significantly decreased antigen uptake from 48 to 72 h was likely caused by antigen digestion and presentation of MigDCs to ResDCs and T/B cells.^[^ ^33^ ^]^

MigDCs were subdivided into CD103^+^ cDC1 and CD11b^+^ cDC2. The dynamic changes of OVA‐Cy5.5 positive populations in cDC2s (Figure 7j; Figure S13d, Supporting Information) and cDC1s (Figure S13e, Supporting Information) exhibited a similar trend as that in MigDCs. However, OVA‐Cy5.5 positive populations in cDC2s were evidently higher than those in cDC1s (≈3 times), indicating that cDC2s were the primary antigen transporters. Beyond DCs, CD11c^−^CD11b^+^F4/80^+^ macrophages (Figure S13f, Supporting Information) demonstrated a similar capability in antigen capture and transport with cDC1s.

Effective antigen cross‐presentation of DCs is necessary for priming CD8^+^ T cells against tumors. As a result, we next evaluated the dynamics of OVA cross‐presentation by DCs in skin dLNs using H‐2Kb/SIINFEKL (MHC I restricted OVA peptide) antibodies (Figure S14, Supporting Information). Following immunization of MANS‐H@OVA, total DCs, MigDCs, and ResDCs all demonstrated the optimal cross‐presentation capability at 48 h with ≈2% MHC I‐OVA^+^ DCs, ≈20% MHC I‐OVA^+^ MigDCs and ≈4% MHC I‐OVA^+^ ResDCs, respectively (Figure S15a–d, Supporting Information), which were in agreement with OVA capture trend. Interestingly, the cDC2 in MigDCs exhibited a similar trend as that in MigDCs (Figure S15e, Supporting Information), however, the frequency of cDC1 presenting MHC I‐SIINFEKL remained increasing over time with the highest population (≈4%) at 72 h (Figure S15f, Supporting Information). The activated DCs were found effectively cross‐presenting SIINFEKL on the surface of CD8^+^ T cells maximally at 48 h (Figure S15g, Supporting Information).

Taken together, MigDCs, particularly cDC2 of MigDCs, effectively sampled antigens delivered by NSs in cooperation with macrophages and transported the captured antigens into dLNs to present ResDCs, which offered promising prerequisite conditions for antigen cross‐presentation and evoking adaptive immune response at the initial stage. Activated DCs are short‐lived, thus cDC1 that cross‐presented antigens at a later stage might complement the capability of activated cDC2 in priming CD8^+^ T cells.

### 2.5 The Potency and Subtypes of Antigen‐Specific Humoral and Cellular Immune Response Determined by the Immunogenically Distinct NS Adjuvants

**Figure 8:**
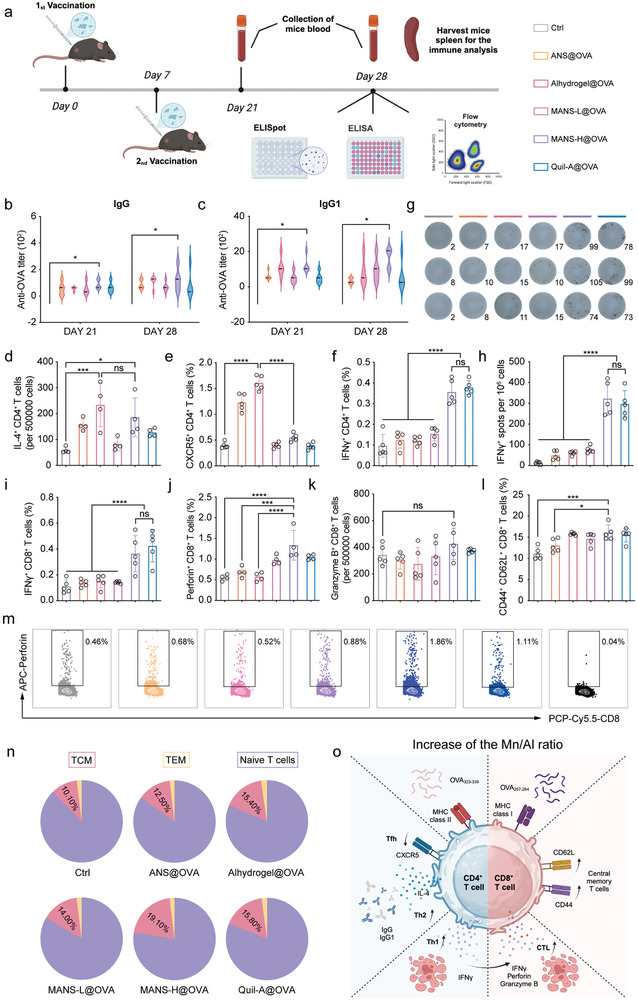
MANS‐H adjuvanted vaccine induces durable and potent humoral and cellular immunity. a) The experiment timeline: C57BL/6 mice were vaccinated twice on day 0 and day 7. Blood samples were collected on day 21 and day 28, and the spleens were harvested at the endpoint for further analysis (created with permission by BioRender). b–c) Serum IgG b) and IgG1c) antibody titers against OVA in vaccinated or control mice (*n =* 5). d–f) The number of OVA_323‐339_specific IL‐4^+^CD4^+^ T cells in 500 000 cells d), the fractions of OVA_323‐339_‐specific CXCR5^+^CD4^+^ T cells e) and OVA_323‐339_specific IFN‐γ^+^ CD4^+^ T cells f) in spleens derived from vaccinated or control mice (*n =* 4 or 5). g‐h) Representative digital photos show the ELISPOT spots of OVA_257‐264_‐specific CD8^+^ T cells secreting IFN‐γ^+^ g) and numbers of OVA‐specific IFN‐γ^+^CD8^+^ T cells per 10^6^ cells h) (*n =* 5). i‐k, m) Flow cytometry analysis shows the proportions of OVA_257‐264_specific IFN‐γ^+^CD8^+^ T cells i), perforin^+^CD8^+^T cells j), and granzyme B^+^CD8^+^ T cells k), and representative dot plots of perforin^+^CD8^+^T cells m) in spleens from vaccinated or control mice (*n =* 4 or 5). l, n) Flow cytometry analysis shows the fractions of CD44^+^CD62L^+^CD8^+^ T cells (TCM, l), and the proportion sums of TCM, TEM, and naïve T cells presented in pie charts in spleens from vaccinated or control mice (*n =* 5). o) A schematic illustration shows the favored subtypes of adaptive immune response mediated by the increased ratio of Mn to Al (created with permission by BioRender). Data are presented as means ± SD. P values were determined by a one‐way ANOVA test. ns, *p >* 0.05, *p < 0.05, ***p < 0.001, ****p < 0.0001.

Given the striking differences between NS adjuvants in immunopotentiation and antigen delivery, we next analyzed the humoral and cellular response using OVA as immunogens. The commercially available adjuvants, Alhydrogel (Th2 bias) and Quil‐A (saponin‐based adjuvants, Th1 bias), were selected for comparison. C57BL/6J mice were subcutaneously injected with OVA combined with adjuvants as indicated in a standard prime‐boost regimen (**Figure** **8**a). Serum samples were collected following boost vaccination for humoral immune response evaluation. All immunized mice demonstrated detectable OVA‐specific IgG at day 21 post‐immunization (Figure 8b; Figure S16a, Supporting Information). MANS‐H indued the highest titer of anti‐OVA IgG, which was comparable with Alhydrogel at day 28. The subclass of IgG1‐type antibodies reflected the involvement of the Th2‐type humoral immune response.^[^ ^34^ ^]^ At day 21, MANS‐H and Alhydrogel induced higher tiers of anti‐OVA IgG1 antibodies than the other three adjuvants (Figure 8c; Figure S16b, Supporting Information). However, MANS‐H outperformed other adjuvants on day 28, suggesting a long‐lasting Th2‐type humoral immune response. Collectively, MANS‐H was the most effective adjuvant in the production of durable humoral immune response, particularly biased Th2‐subtype, followed by Alhydrogel and then ANS/MANS‐L/Quil‐A.

To analyze OVA‐specific T cell immune response, splenocytes derived from immunized mice were re‐stimulated by SIINFEKL (OVA_257‐264_, MHC class I restricted OVA peptide) and ISQAVHAAHAEINEAGR (OVA_323‐339_, MHC class II‐restricted OVA peptide) and analyzed by enzyme‐linked immune absorbent spot (ELISPOT) assay and flow cytometry analysis. MANS‐H and Alhydrogel significantly enhanced the frequencies of OVA‐specific CD4^+^ T cells producing IL‐4 cytokine (Figure 8d; Figure S17a, Supporting Information), implying a high production of Th2‐ cells. By contrast, ANS, MANS‐L, and Quil‐A adjuvants showed relatively low frequencies of OVA‐specific Th2 cells. Interestingly, ANS and Alhydrogel drove dramatically enhanced differentiation of Th cells into CXCR5^+^CD4^+^ Tfh in comparison to MASN‐L, MASN‐H, and Quil‐A adjuvants (Figure 8e), indicating that Al element favored Tfh‐biased immunity. By contrast, MANS‐H and Quil‐A exhibited significantly higher proportions of OVA‐specific IFN‐γ^+^CD4^+^ Th1 subtype of T cell immunity than other adjuvants (Figure 8f), implying that Mn element and saponin supported Th1 T cell immune response. MANS‐H and Quil‐A adjuvanted OVA vaccines elicited markedly higher OVA‐specific CD8^+^ T cells producing IFN‐γ than the other groups, evidenced by the significant spots in the IFN‐γ ELISPOT assay (Figure 8g,h).

To assess the cytolytic activity of OVA‐specific effector CD8^+^ T cells, the secretion levels of cytotoxic molecules, including IFN‐γ cytokine, granzyme B, and pore‐forming perforin in lytic granules,^[^ ^10^, ^35^ ^]^ were analyzed upon restimulation. The flow cytometry results revealed that the frequencies of OVA‐specific CD8^+^ T cells producing IFN‐γ were prominent in MANS‐H and Quil‐A adjuvanted vaccines (Figure 8i; Figure S17b, Supporting Information), which were consistent with ELISPOT analysis. MANS‐H adjuvant displayed the highest proportion of OVA‐specific CD8^+^ T cells secreting perforin (Figure 8j,m). However, a moderate increase was observed in the population of granzyme B^+^CD8^+^ T cells in MANS‐H adjuvanted vaccine (Figure 8k). We also examined memory phenotypes of T cells in the spleen. Vaccination of MANS‐H@OVA but not other vaccines resulted in markedly increased CD44^+^CD62L^+^CD8^+^ T cells (T central memory, TCM, Figure 8l). The frequencies of other phenotypes of CD44^+^CD62L^−^CD8^+^ T cells (T effector memory, TEM) and CD44^−^CD62L^+^CD8^+^ T cells (naïve T cells) were not obviously altered in all groups (Figure 8n; Figure S18a,b, Supporting Information).

In summary, the increase of Mn/Al ratio favored the induction of durable Th2‐type humoral immune response and heterogeneous subsets of the antigen‐specific cellular immune response, including Th2, Th1, and multiple functional CD8^+^ T cells, and long‐lived CD8^+^ TCM, but suppressed the production of Tfh subtype of T cell immunity. MANS‐H adjuvanted vaccine induced multifaceted, effective, and long‐lasting humoral and cellular immunity, particularly polyfunctional CD8^+^ T cells, predicting superior therapeutic effect in eliminating cancer by comparing to other NS adjuvants (Figure 8o).

### 2.6 Murine Melanoma Regression by MANS‐H Adjuvanted Vaccine Therapy

**Figure 9:**
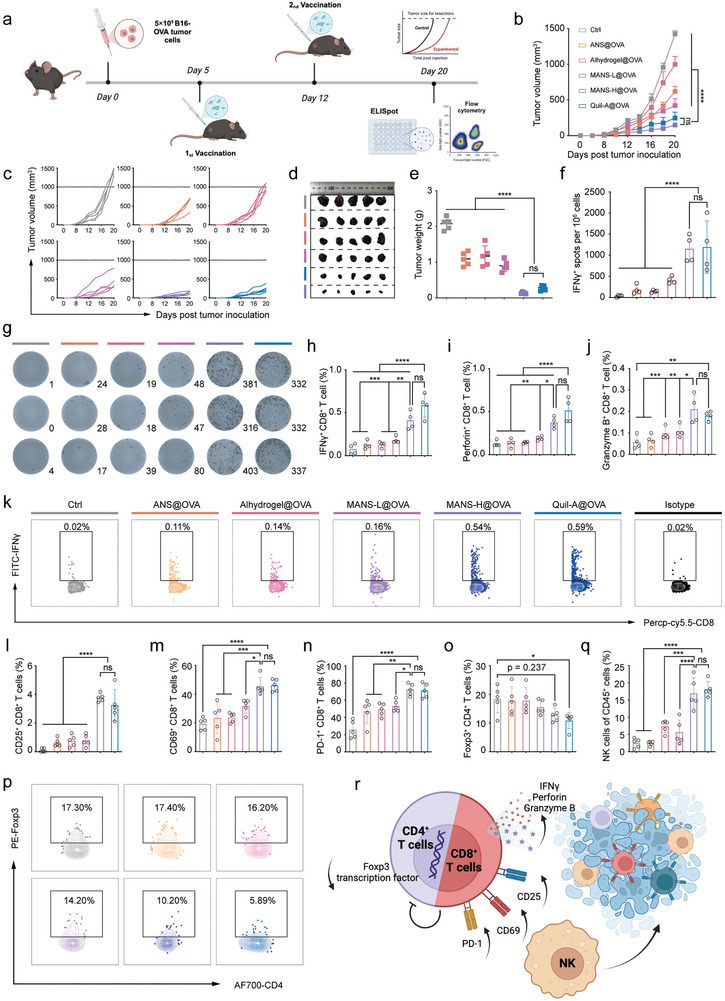
MANS‐H adjuvanted OVA vaccine significantly suppresses B16‐OVA tumor growth by boosting polyfunctional T cell immune response. a) The experimental timeline of the therapeutic regimen of the B16‐OVA melanoma model: C57BL/6J mice were subcutaneously inoculated with 5 × 10^5^ B16‐OVA cells on day 0 and then vaccinated twice with weekly intervals on day 5 and day 12 (created with permission by BioRender). b) Average and c) individual B16‐OVA tumor growth volume curves of immunized and unimmunized mice (*n =* 5). d) Digital photos and e) weights of tumor samples harvested at the endpoint (*n =* 5). f) ELISPOT analysis shows the numbers of OVA‐specific IFN‐γ^+^CD8^+^ T cells per 10^6^ splenocytes and g) the representative digital photos of CD8^+^ T cells secreting IFN‐γ dots in the spleen after restimulation with OVA_257‐264_ peptides (*n =* 4). h–k) Flow cytometry analysis shows the proportions of h) OVA‐specific IFN‐γ^+^CD8^+^ T cells, i) perforin^+^CD8^+^ T cells and j) granzyme B^+^ CD8^+^ T cells, and representative dot plots of k) IFN‐γ^+^CD8^+^ T cells in spleens (*n =* 4). l–p) Flow cytometry analysis summarizes the percentages of B16‐OVA tumor‐infiltrating l) CD25^+^CD8^+^ T cells, m) CD69^+^CD8^+^ T cells, n) PD‐1^+^CD8^+^ T cells, o) Foxp3^+^CD4^+^ Tregs and q) NK cells, and p) representative dot plots of Foxp3^+^CD4^+^ T cells (*n =* 5). r) A schematic illustration shows the cytolytic functions of polyfunctional effector CD8^+^ T cells and NK cells mediate potent anti‐tumor effect (created with permission by BioRender). Data are presented as means ± SEM (B) or SD (e,f, h–j, l–o, and P) (*n =* 5). P values were determined by two‐way ANOVA, Tukey's multiple‐comparison test (b), or one‐way ANOVA, Tukey's multiple‐comparison test (e,f, h–j, l–o, and p). n.s., not significant; ^*^ *p <* 0.05; ^**^ *p <* 0.01; ^***^ *p <* 0.001; ^****^ *p <* 0.0001.

To examine the immunotherapeutic potency of NS‐adjuvanted OVA vaccines, we established a therapeutic regimen in an OVA‐expressing murine melanoma B16F10 (B16‐OVA, Figure S19a, Supporting Information) model, in which C57BL/6J mice were inoculated with B16‐OVA cells on day 0 and then received prime‐boost vaccination at day 5 and day 12 respectively (**Figure** **9**a). All vaccinated groups exhibited significant suppression of B16‐OVA tumor growth (Figure 9b–e), while the suppression effect of MANS‐H@OVA and Quil‐A@OVA was comparable and the most pronounced. During the treatment, there was no obvious weight loss among all groups of mice (Figure S19b, Supporting Information), suggesting that vaccination treatment was well tolerated. We next characterized the magnitude and functionality of antigen‐specific CD8^+^ T cells in the spleen, which are key mediators for tumor elimination.^[^ ^36^ ^]^ To this end, splenocytes were re‐stimulated by SIINFEKL and analyzed by ELISPOT assay and flow cytometry analysis. In comparison with unvaccinated or vaccinated mice with Alhydrogel@OVA, MANS‐L@OVA, or ANS@OVA, mice vaccinated with MANS‐H@OVA elicited significantly higher OVA‐specific CD8^+^ T cells producing IFN‐γ, proved by the substantial spots in the IFN‐γ ELISPOT assay (Figure 9f,g). A similar magnitude of OVA‐specific CD8^+^ T cells was observed in the Quil‐A@OVA group. The flow cytometry results revealed a similar trend in the frequency of OVA‐specific IFN‐γ^+^CD8^+^ T cells (Figure 9h,k; Figure S20a, Supporting Information). Beyond the production of IFN‐γ, OVA‐specific CD8^+^ T cells elicited by MANS‐H@OVA and Quil‐A@OVA were capable of secreting markedly higher levels of perforin (Figure 9i; Figure S20b, Supporting Information) and cytolytic pore‐forming proteins of granzyme B (Figure 9j) than other groups. Altogether, these results suggested that the antigen‐specificity, high number, and multifaceted effector functionality of vaccination‐induced CD8^+^ T cells supported the observed potent anti‐tumor effect of MANS‐H@OVA and Quil‐A@OVA.

We next examined the frequency of immune cells and phenotypes of tumor‐infiltrating lymphocytes (TILs) to explore the immuno‐features of the tumor microenvironment (Figure S21, Supporting Information). A substantial increase in tumor‐infiltrating CD45^+^ immune cells was observed in mice vaccinated with MANS‐H@OVA and Quil‐A@OVA compared with unimmunized mice (Figure S22a, Supporting Information), and a trend toward increased CD45^+^ immune cells in other immunized groups. MANS‐H@OVA and Quil‐A@OVA significantly promoted the frequency of CD8^+^ TILs (Figure S22b, Supporting Information), while suppressing CD4^+^ TILs (Figure S22c, Supporting Information), resulting in enhanced ratios of CD8^+^/CD4^+^ TILs (Figure S21d, Supporting Information). MANS‐H@OVA and Quil‐A@OVA exhibited dramatically enhanced distinct subpopulations of CD8^+^ TILs with activation markers of CD25 (Figure 9l; Figure S23a, Supporting Information), CD69 (Figure 9m; Figure S23b, Supporting Information) and PD‐1 (Figure 9n; Figure S23c, Supporting Information). CD25^+^ TILs and PD‐1^+^ TILs that are terminally differentiated are also defined as exhausted and dysfunctional phenotypes of TILs.^[^ ^37^ ^]^ In addition, the infiltration of regulatory T cells (Tregs, Foxp3^+^CD4^+^ T cells) was greatly suppressed in tumors when mice received MANS‐H@OVA or Quil‐A@OVA, but not other immunization treatments (Figure 9o,p). Interestingly, the proportions of tumor‐infiltrating NK cells were strikingly raised in the MANS‐H@OVA and Quil‐A@OVA group (Figure 9q; Figure S23d, Supporting Information), which also contributed to their outperformed tumor‐killing effect. Tumor tissue staining images revealed decreased proliferative activity and increased apoptosis and necrosis (Figure S24, Supporting Information) in tumor samples from mice vaccinated with MANS‐H@OVA or Quil‐A@OVA.

Collectively, MANS‐H displayed comparable potency compared with the commercial cellular response adjuvant Quil‐A in triggering the production of antigen‐specific CD8^+^ T cells with multifaceted functionality, which resulted in dramatically increased cytolytic effector CD8^+^ TILs and NK cells while suppressed Tregs infiltrated in tumor for superior anti‐tumor efficacy (Figure 9r).

### 2.7 Tumor Suppression in Preclinical Murine Human Papillomavirus (HPV) Positive Tumor Models

**Figure 10:**
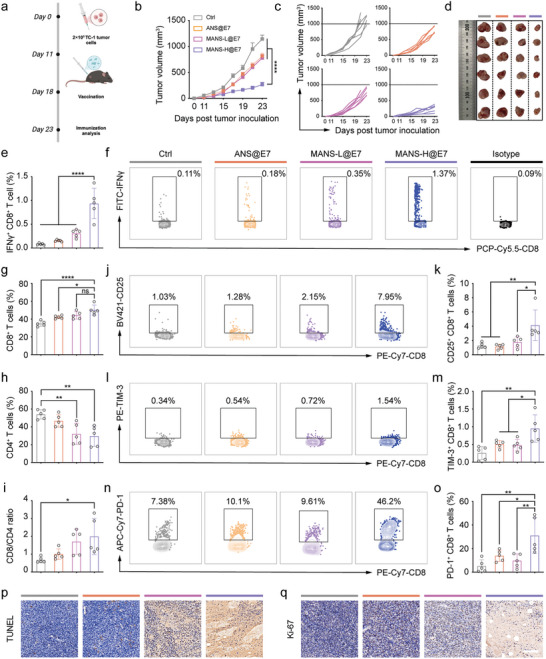
MANS‐H adjuvanted E7 vaccine remarkably regresses the HPV E7‐expressing tumor growth. a) A scheme shows the experimental timeline: C57BL/6J mice were subcutaneously inoculated with 2 × 10^5^ TC‐1 cells on day 0, and then vaccinated with diverse E7 vaccine formulations on day 11 and day 18 (created with permission by BioRender). b) Average and c) individual TC‐1 tumor growth curves of unimmunized and immunized mice (*n =* 6). d) Digital photos of tumor samples harvested at the endpoint on day 23 (*n =* 6). e,f) The splenocytes were re‐stimulated with E7 peptide for the detection of E7‐specific IFN‐γ^+^ CD8^+^ T cells. e) Flow cytometry analysis shows the percentages and f) the representative dot plots of E7‐specific IFN‐γ^+^CD8^+^ T cells (*n =* 5). g‐o) Flow cytometry analysis shows the proportions of g) CD8^+^ T cells, h) CD4^+^ T cells and the ratios of i) CD8^+^/CD4^+^ T cells in CD45^+^ cells, the representative dot plots and bar charts presenting the proportions of j,k) CD25^+^CD8^+^ T cells, l,m) TIM‐3^+^CD8^+^ T cells and n,o) PD‐1^+^CD8^+^ T cells pre‐gated on CD8^+^ T cells in TC‐1 tumors derived from unimmunized and immunized mice (*n =* 5). p,q) Representative immunofluorescence images of tumor tissue sections stained with p) TUNEL or q) Ki‐67. Scale bars: 100 µm. Data are presented as means ± SEM (b) or SD (e, g–i, k, m and o). P values were determined by two‐way ANOVA, Tukey's multiple‐comparison test (b), or one‐way ANOVA, Tukey's multiple‐comparison test (e, g–i, k, m and o). n.s., not significant, ^*^ *p <* 0.05; ^**^ *p <* 0.01; ^****^ *p <* 0.0001.

Instead of model antigens, we incorporated our MANS‐H adjuvant with an HPV16‐E7 antigen (RAHYNIVTF) and evaluated the therapeutic efficacy in a preclinical model of HPV16‐positive tumor TC‐1 in C57BL/6J mice. In a therapeutic regimen, C57BL/6J mice were subcutaneously injected with TC‐1 cells on day 0 and received two doses of therapeutic adjuvanted E7 vaccines on day 11 and 18 respectively (**Figure** **10**a). Compared with the groups of Ctrl, ANS@E7, and MANS‐L@E7, MANS‐H@E7 vaccine significantly inhibited TC‐1 tumor growth (Figure 10b–d; Figure S25a, Supporting Information). There was no obvious weight loss during the experimental period cross all groups (Figure S25b, Supporting Information), suggesting the well‐tolerated safety profiles of these NS‐adjuvanted vaccines.

We next sought to understand the anti‐tumor activity of the MANS‐H@E7 vaccine by examining the E7‐specific CD8^+^ T cell immune response induced by vaccination. The flow cytometry results demonstrated that the MANS‐H@E7 vaccine induced the highest proportion of E7‐specific IFN‐γ^+^CD8^+^ T cells among all groups (Figure 10e,f; Figure S26, Supporting Information), implying a potent cytotoxic function against the tumor. We next assessed the subsets of TILs in TC‐1 tumor. As expected, the MANS‐H@E7 vaccine group substantially increased the fraction of CD8^+^ TILs (Figure 10g; Figure S27, Supporting Information) and reduced the proportion of CD4^+^ TILs (Figure 10h; Figure S27, Supporting Information), leading to a higher ratio of CD8^+^/CD4^+^ TILs (Figure 10i) than other three groups. We next analyzed the phenotypes of CD8^+^ TILs (Figure S28, Supporting Information). The results displayed a remarkable rise in the effector functional CD25^+^CD8^+^ TILs in mice vaccinated with MANS‐H@E7 but not ANS@E7 or MANS‐L@E7 (Figure 10j,k). T cell immunoglobulin mucin receptor 3 (TIM‐3) and PD‐1 are usually defined as inhibitor markers, but also termed effector‐like markers when TILs are non‐terminally exhausted.^[^ ^37^ ^]^ Interestingly, the MANS‐H@E7 vaccine dramatically promoted the frequencies of TIM‐3^+^CD8^+^ TILs and PD‐1^+^CD8^+^ TILs (Figure 10l–o). Further analysis of the proliferative activity of these subsets of CD8^+^ TILs would define their effector‐like or exhausted phenotypes, thereby determining the necessity of the combination of anti‐PD‐1 or anti‐TIM‐3 monoclonal antibodies for enhanced cancer treatment. In addition, the analysis of the proliferation (Figure 10p), apoptosis and necrosis (Figure 10q; Figure S29, Supporting Information) of TC‐1 tumor samples suggested the strongest anti‐tumor efficacy of MANS‐H@E7 vaccine, which was in line with the trend of tumor growth. Immunofluorescent staining results revealed a substantially increased number of CD8‐positive T cells infiltrated in tumor sites from mice receiving vaccination with MANS‐H@E7, which was consistent with the flow cytometry results.

### 2.8 In Vivo Assessments of Biodistribution and Biosafety Profiles of MANS‐H

To explore the biodegradability of MANS‐H in vivo, mice were subcutaneously injected with MANS‐H@OVA‐Cy5.5 and then imaged by the in vivo imaging systems (IVIS) at different timepoints. The results showed that fluorescence signals of OVA‐Cy5.5 gradually declined over the monitored period while there were still some signals at day 14 (Figure S30, Supporting Information), suggesting that the vaccine formulation was readily degraded and retained at the injection site for more than 14 days. The biosafety of MANS‐H was further evaluated over a period of 14 days. The blood routine test and hepatorenal function indices (Figure S31a,b, Supporting Information) revealed that there was no significant difference between the control and MANS‐H immunized groups. The histology studies demonstrated that there was no evident lesion toxicity in the major organs and no apparent inflammation was observed at the injection site either at day 7 or 14 post‐vaccination (Figure S31c, Supporting Information). These results demonstrated satisfactory biosafety of MANS‐H in vivo.

According to the guidelines provided by the World Health Organization, pharmacokinetic studies of vaccines are not considered a prerequisite for their clinical approval. However, there are a few reports exploring the pharmacokinetics, half‐life, and clearance mechanism of inorganic adjuvants.^[^ ^34^, ^38^ ^]^ These results suggested that the half‐life of Al ions released from intramuscularly injected Alum adjuvants was ≈28 days in rabbits,^[^ ^38^ ^]^ while the silica adjuvants subcutaneously injected were possibly excreted via the kidneys.^[^ ^34^ ^]^ Our MANS‐H adjuvant might follow similar features with these inorganic adjuvants, which would be validated in our future work.

## 3 Conclusion

Immunotherapy aims to train the host to generate potent and durable immunity against cancer, which has revolutionized cancer treatments, particularly antigen‐specific cancer immunotherapy.^[^ ^39^ ^]^ Despite the great success of immune checkpoint blockade (ICB) therapy in the clinic, only a small fraction of cancer patients can benefit from ICB (≈10–30%). Therapeutic vaccines are an alternative strategy to efficiently eradicate malignant cells by orchestrating antigen‐specific T cells.^[^ ^40^ ^]^ DCs are the largest and most crucial subsets of APCs, which are responsible for the capture, process, and presentation of various antigens. In the context of cancer immunotherapy, priming antigen‐specific CD8^+^ T cells by cross‐presenting DCs is the key to regressing tumors. The cytolytic functions and numbers of CD8^+^ T cells primarily rely on the positive signals from DCs stimulated by adjuvants and help signals of CD4^+^ Th cells. The activity of adjuvants determines the maturation levels of DCs and subtypes of T‐cell immunity. MANS‐H constructed in this work demonstrated sufficient stimulation of cDC2 and cross‐presenting CD103^+^/CD8^+^ cDC1, which supported the observation of a markedly high magnitude of effector and memory phenotypes of CD8^+^ T cells and durable humoral immunity, significantly suppressing tumor growth. In addition, accumulated evidence has revealed the significance of incorporating appropriate immunostimulants and antigens in structuring nano‐vaccines for sufficient co‐transport of antigens and stimulators, and augmented effectiveness and functions of CD8^+^ T cells.^[^ ^9^ ^]^ In addition to chemical components, physical architecture is also a key determining factor, driving the immune response to rational structuring vaccines.^[^ ^1^, ^41^ ^]^ Current emerging strategies mainly focus on zero‐ or three‐dimensional architectures to regulate vaccine biodistribution and immune modulation, with few reports on 2D particulate vaccines.^[^ ^16^ ^]^ Our results revealed that Al either in the form of NS or salts drove the production of antigen‐specific Tfh immune response, while the incorporation of Mn into ANS shaped the immune response to Th1 and CD8^+^ T immunity and long‐lasting antibodies, which were consistent with the observations in the comparison between Alum and Mn salts.^[^ ^9^ ^]^ Differently, ANS containing high contents of Mn showed a similar level of Th2 immune response compared to Alum, which might be contributed by the hybrid components of Al and Mn. Additionally, the potent therapeutic effectiveness of MANS adjuvanted cancer vaccines was evidenced in the therapeutic regimens that were not examined in the previously reported Mn salt adjuvanted vaccine.^[^ ^9^ ^]^

It has been reported that the hyperactive DCs stimulated long‐lived T‐cell responses, which are critically important for the complete eradication of established tumors.^[^ ^42^ ^]^ Therefore, to take the encouraging results from the preclinical model forward (Figure 10), it necessitates assessing T cell memory immune responses of MANS‐H adjuvanted HPV16‐E7 vaccine in long‐term survivability (more than 150 days in murine therapeutic settings) and tumor rechallenge models. These in‐depth preclinical studies will uncover additional factors that might impact the therapeutic efficacy of our adjuvant and cancer vaccines.

In conclusion, we have uncovered the immune‐potentiating qualities of 2D NS containing immunologically different MnAl. MANS‐H has shown remarkable proficiency in stimulating various subsets of innate immune cells and triggering a highly heterogeneous and functional tumor‐specific immunity. This led to superior tumor regression when combined with model or viral antigens in aggressive malignant melanoma and preclinical HPV^+^ murine cancer models. These results highlight its potential for clinical use in developing therapeutic cancer vaccines.

## 4 Experimental Section

### Chemicals and Biological Agents

Aluminum acetylacetonate was purchased from J&K Scientific Ltd. KMnO_4_, toluene, and sodium chloride were purchased from Sinopharm Chemical Reagent Co., Ltd. IFN‐β and IL‐1β ELISA kits were purchased from MULTISCIENCES (LIANKE) Biotech, Co., Ltd. DNase I was purchased from Nanjing Keygen Biotech. Collagenase D was purchased from Roche. OVA, OVA_257‐264_ (SIINFEKL), OVA_323‐339_ (ISQAVHAAHAEINEAGR), Quil‐A, and Alhydrogel were purchased from Invivogen. OVA‐Cy5.5 was purchased from XiAn QIYUE Biotech, Co Ltd. HPV16 E7 peptide (RAHYNIVTF) was purchased from GL Biochem, Ltd., Shanghai.

### Mice and Cell Lines

Six to eight weeks aged C57BL/6J mice were purchased from Shanghai Model Organisms Centre Inc. All experimental procedures were performed under the policies of the National Ministry of Health with the approval of the Shanghai University Animal Ethics Committee (approved ID: YS‐2023‐134). B16‐OVA and TC‐1 cells were maintained in a humidified atmosphere (37 °C, 5% CO_2_).

### Synthesis of NaCl Crystals

Typically, the equivalent volume of ethanol was added into the saturated aqueous NaCl solution (37 g NaCl per 100 mL deionized H_2_O) and kept at static conditions for 30 min for recrystallization. The slurry‐like sediment was harvested by filtration through filter papers and washed with absolute ethanol. The wet NaCl templates were filled into 2 mL centrifuge tubes for condensing under 3000 rpm for 5 min, followed by a drying process at 70 °C oven for 4 h.

### Synthesis of ANS, MANS‐L, and MANS‐H

ANS was synthesized by a hard template method. Briefly, aluminum acetylacetonate (768 mg) was dissolved in toluene (40 mL) for 30 min under sonication. The precursor supernatant was collected with centrifugation (9000 rpm, 10 min), and then was added to hard NaCl templates with a ratio of 30% (V/W, 300 µL precursor solution per gram of templates). Standing for 10 min allowed the precursor solution to wet through the surface of NaCl templates filled in centrifuge tubes, which were kept in a humidity chamber (Shanghai Yiheng Technology Instrument Co., Ltd.) with 85% relative humidity and 25 °C temperature for 48 h. Finally, ANS was obtained by washing with water 3 times to remove NaCl templates and washing with absolute ethanol once to remove incomplete reacted precursors.

MANS‐L and MANS‐H were prepared by doping Mn on ANS. In particular, ANS (5 mg) was suspended in water (5 mL) to prepare the solution with a concentration of 1 mg mL^−1^. Then, 4 mL KMnO_4_ solution with a concentration of 1.3 or 0.1625 mg mL^−1^ was added in ANS (5 mL, 1 mg mL^−1^) under stirring (300 rpm h^−1^, in dark) for 1 h for the synthesis of MANS‐L and MANS‐H, respectively. MANS‐L and MANS‐H samples were obtained with centrifugation (15 000 rpm, 10 min) and washed 3 times with water.

### Characterization of Micro‐nanosheets

NSs were suspended in anhydrous ethanol for a simple ultrasound followed by dropping on the carbon‐covered Cu grid, which was used for the TEM imaging or energy dispersive X‐ray spectroscopy (EDS) analysis (JEOL 1400 or JEOL 2100F). Additionally, NSs were dried at 70 °C in the drying oven for 6 h for the XPS analysis (Escalab 250Xi, Thermo Scientific) and Fourier transformed infrared (FTIR) analysis (Nicolet iS50, Thermo Scientific). NSs were suspended in PBS and measured by Zetasizer Nano ZSE for the detection of zeta potential (25 °C). Few NSs were soluble in aqua regia for 24 h which was used for the analysis of ICP (PERKINE 7300DV, Perkin Elmer).

### BMDC and Macrophage Culture

The extraction and culture of BMDCs were according to the previously reported method.^[^ ^43^ ^]^ In brief, bone marrow cells were collected from the femur and tibia of naive C57BL/6J mice and seeded in a 100 mm culture dish with RPMI1640 complete medium supplemented with 100 ng mL^−1^ IL‐4 and 200 ng mL^−1^ GM‐CSF. On day 3 and day 6, the culture medium was replaced with a fresh BMDC medium. On day 7, BMDCs were harvested for subsequent experiments. For bone marrow macrophages, bone marrow cells were cultured in DMEM complete medium with 200 ng mL^−1^ M‐CSF, while the remaining procedures remained unchanged.

### BMDC Activation

Briefly, BMDCs (5 × 10^5^ cells per tube, 1 mL) were seeded in a flow cytometry caped tube (BD Falcon), followed by the treatment of PBS or NSs (ANS, MANS‐L or MANS‐H) (15 µL, 1 mg mL^−1^). After 24 h incubation in a cell incubator, the cell pellets were washed with cold PBS followed by collection by centrifugation (350 g, 4 °C, 5 min) for the further staining of flow cytometry analysis.

### Intracellular ROS Detection

BMDCs (5 × 10^5^ cells per tube, 0.5 mL) were seeded in a flow cytometry caped tube and then treated with PBS or NSs (15 µL, 1 mg mL^−1^). After incubation for 10 h, the 0.5 mL DCF‐DA solution (40 µm, Sigma) was added for 20 min incubation. Then cell pellets were washed with cold PBS followed by collection by centrifugation (350 g, 4 °C, 5 min) for the further staining of flow cytometry analysis. In another experiment, BMDCs (5 × 10^5^ per well, 0.5 mL) were seeded in a 24‐well plate containing round coverslips and cultured for 24 h. After a 10 h co‐culturation with PBS or NSs (15 µL, 1 mg mL^−1^), the 0.5 mL DCF‐DA solution (40 µm) was added for 20 min incubation (in dark). Following two times wash with cold PBS, 4,6‐diamidino‐2‐phenylindole (DAPI, Beyotime) was dropped on coverslips for intracellular ROS detection by confocal microscopy (LSM710, Zeiss).

### OVA Antigen Loading Capacity

The loading capacity of ANS, MANS‐L, and MANS‐H NSs with OVA‐Cy5.5 was estimated by detecting fluorescence intensity using a microplate reader. In detail, NS (1 mL, 6 mg mL^−1^) was mixed with poly (allylamine hydrochloride)^[^ ^32^ ^]^ (2 mL, 20 mg mL^−1^) for stirring at 600 rpm for 1 h at room temperature. After that, the NS@PAH was collected by centrifugation (14 000 rpm, 10 min) and re‐suspended in 1 mL PBS. OVA‐Cy5.5 (1 mL, 600 µg mL^−1^) was added in NS@PAH solution (1 mL, 6 mg mL^−1^) under stirring at 600 rpm for 0.5 h at 4 °C (in dark). The supernatant containing excess OVA‐Cy5.5 was collected by centrifugation (15 000 rpm, 20 min). The fluorescence intensity of 100 µL of supernatant or standard solutions placed in a 96‐well plate was measured by a microplate reader (fluorescence excitation peak at 656 nm and emission peak at 700 nm. VARIOSKAN LUX, Thermo Scientific).

### Cellular Uptake of NS Adjuvanted OVA‐Cy5.5 Vaccines in BMDCs

Briefly, OVA‐Cy5.5 was loaded with three NSs as previously described. BMDCs (5 × 10^5^ cells per tube, 1 mL) were seeded in a flow cytometry capped tube. Then PBS, free OVA‐Cy5.5, and three NSs adjuvanted OVA‐Cy5.5 vaccines (15 µL, 1 mg mL^−1^) were added respectively and further cultured for 6 h. Following the wash with cold PBS, the cell pellets were obtained for flow cytometry staining and analysis or staining with iFluorTM 488 phalloidin and DAPI for confocal imaging (STELLARIS 8, Leica).

### In vivo Biodistribution of OVA‐Cy5.5 and Cross Presentation of OVA Mediated by NS Adjuvants

As mentioned above, mice were vaccinated with PBS, free OVA‐Cy5.5 or three NSs (300 µg) adjuvanted OVA‐Cy5.5 or OVA vaccines. After 24, 48, or 72 h, the skin‐dLNs were harvested respectively and were digested according to the established method (see below in single cell preparation) followed by staining for flow cytometry analysis. To investigate the degradation of NS, C57BL/6J mice were subcutaneously injected with MANS‐H adjuvanted OVA‐Cy5.5 vaccine and visualized by IVIS spectrum system at different time points (day 0, day 7, and day 14).

### Biosafety Evaluation

The biosafety evaluation was performed in C57BL/6J mice subcutaneously injected with 300 µg MANS‐H. The blood samples, major organs, and skin samples at the injection site were harvested on days 7 and 14 post‐injection. A blood routine test was performed to analyze the levels of red blood cells (RBC), white blood cells (WBC), platelets (PLT), and hemoglobin (HGB). After centrifugation at 12 000 rpm for 20 min, the serum samples were obtained for biochemical analysis of the levels of alanine aminotransferase (ALT), aspartate aminotransferase (AST), creatinine (CREA), and UREA. The tissue samples were stained with hematoxylin and eosin for histology studies.

### Tumor Cell Culture and Development

Briefly, B16‐OVA and TC‐1 cells were injected subcutaneously into the abdomen of the mice with the indicated cell concentration in the Figures. Tumor size was measured every two days and tumor volume was estimated as length × width^2^/2. When tumor volume reached 1500 mm^3^, animals were sacrificed.

### Single‐cell Suspension Preparation for Flow Cytometry Analysis

Acquired skin samples (−1 cm^2^, in the administration site), skin‐dLNs, and tumor tissues were chopped thoroughly and incubated with a solution containing DNase I (0.2 mg mL^−1^) and collagenase D (1 mg mL^−1^) for 1.5 h,1 h, and 0.5 h (37 °C) respectively. All cells were filtered through the 70 µm cell strainers (Biosharp) followed by being re‐suspended for the next flow cytometry staining. Spleens were harvested at the indicated point in Figures, which were mechanically disrupted through 70 µm cell strainers. Following washed by FACS buffer and lysed with ACK lysis buffer, splenocytes were re‐suspended for further flow cytometry staining. For antigen‐specific T cell detection, splenocytes were seeded in 96‐well plates followed by the addition of OVA_257‐264_, OVA_323‐339_, or E7 peptides (10 µg mL^−1^) for 2 h stimulation. The solution included GolgiPlug (1:1000, BD Biosciences) was added for another 4 h stimulation. Then, splenocytes were collected and washed for flow cytometry staining.

### ELISPOT Analysis of Antigen‐specific CD8^+^ T Cells

Splenocytes (2.5 × 10^5^ cells per well, 50 µL) were seed in a definite 96‐well plate (Millipore, Merck), which was cultured with anti‐IFN‐γ antibody (14‐7313‐85, eBioscience) overnight at 4 °C in advance. Then the OVA_257‐264_ or E7 peptides (10 µg mL^−1^) were added for another 20 h incubation in a cell incubator. After removal of cells, biotinylated Ab against IFN‐γ (13‐7312‐85, eBioscience) was added for 4 h incubation at room temperature in the dark followed by the addition of HRP‐conjugated streptavidin (A3151, Sigma‐Aldrich) for incubation 45 mins. After washing several times, PBS solution containing UREA and DAB tablets (D0426, Sigma‐Aldrich) was added to the holes of the plate. Lastly, spots were recorded and counted by an ELISPOT plate reader (AID EliSpot Reader System, Germany).

### Cells Staining of Flow Cytometry Analysis

To avoid non‐specific binding of antigens and to separate dead‐live cells, all cell suspensions were stained first with anti‐CD16/32 and LIVE/DEAD Fixable Aqua (Invitrogen). The skin cells were stained with a mixture of antibodies containing anti‐F4/80 (FITC), anti‐TCR‐β (FITC), anti‐CD45 (Percp‐Cy5.5), anti‐CD11b (Pacific blue), anti‐EpCAM (APC), anti‐MHC II (APC‐Cy7), anti‐CD103 (PE), anti‐CD11c (PE‐Cy7), anti‐Ly6G (Alexa Fluor 700), and anti‐Ly6C (Brilliant Violet 510). The dLNs cells were stained with a mixture of antibodies containing anti‐CD80/anti‐CD19/anti‐TCR‐β/anti‐CD3 (FITC), anti‐CD8a (PerCP‐Cy5.5), anti‐EpCAM/anti‐CD11b/anti‐CD8a (APC), anti‐MHC II/anti‐ H2Kb to SIINFEKL (APC‐Cy7), anti‐CD103 (PE), anti‐CD11c (PE‐Cy7), anti‐CD11b (Alexa Fluor 700) and anti‐CD86/anti‐F4/80/anti‐MHC II (Pacific blue). The B16‐OVA or TC‐1 tumor cells were stained with a mixture of antibodies containing anti‐CD45 (Percp‐Cy5.5), anti‐TCR‐β (FITC), anti‐CD4 (AF700), anti‐CD25 (Brilliant Violet 421), anti‐CD69/anti‐NK 1.1/CD8a (PE‐Cy7), anti‐CD8a/anti‐CD3/anti‐TIM‐3 (PE) and anti‐PD‐1 (APC‐Cy7). Following Fixation/Permeabilization (Thermo Fisher) for 45 mins, tumor‐infiltrating lymphocytes were stained with anti‐Foxp3 (Alexa Flour 647) or isotype antibody. The splenocytes were stained with a mixture of antibodies containing anti‐TCR‐β (Pacific blue), anti‐CD8a (PCP‐Cy5.5), anti‐CD4 (PE‐Cy7), anti‐CD62L (PE), anti‐CXCR5 (APC‐Cy7) and anti‐CD44/anti‐CD4 (Alexa Fluor 700). Following Fixation/Permeabilization for 45 mins, splenocytes were stained with a master mix of antibodies containing anti‐IFN‐γ (FITC), anti‐Perforin/anti‐IL‐4 (APC), and anti‐Granzyme B (APC‐Cy7) or corresponding isotype antibodies for intracellularly cytokines detection. At last, all stained cells were resuspended in FACS buffer which was analyzed by flow cytometer (BD LSRFortessa). All detailed information on antibodies can be found in Table S1 (Supporting Information). Flow cytometry data were analyzed by FlowJo 10.8.1 or Kaluza software.

### Statistical Analysis

Statistical data were analyzed by GraphPad Prism 9.5.1. and detailed analysis methods were outlined in the indicated Figures.

## Conflict of Interest

The authors declare no conflict of interest.

## Supporting information

Supporting Information

## Data Availability

The data that support the findings of this study are available from the corresponding author upon reasonable request.

## References

[1] a) B. Pulendran , P. S. Arunachalam , D. T. O'Hagan , Nat. Rev. Drug Discovery 2021, 20, 454; 33824489 10.1038/s41573-021-00163-y PMC8023785

[2] H. Ren , W. Jia , Y. Xie , M. Yu , Y. Chen , Chem. Soc. Rev. 2023, 52, 5172. 37462107 10.1039/d2cs00848c

[3] B. F. Haynes , K. Wiehe , P. Borrrow , K. O. Saunders , B. Korber , K. Wagh , A. J. McMichael , G. Kelsoe , B. H. Hahn , F. Alt , G. M. Shaw , Nat. Rev. Immunol. 2022, 23, 142. 35962033 10.1038/s41577-022-00753-w PMC9372928

[4] P. Marrack , A. S. McKee , M. W. Munks , Nat. Rev. Immunol. 2009, 9, 287. 19247370 10.1038/nri2510 PMC3147301

[5] a) P. Matzinger , Annu. Rev. Immunol. 1994, 12, 991; 8011301 10.1146/annurev.iy.12.040194.005015

[6] a) V. Hornung , F. Bauernfeind , A. Halle , E. O. Samstad , H. Kono , K. L. Rock , K. A. Fitzgerald , E. Latz , Nat. Immunol. 2008, 9, 847; 18604214 10.1038/ni.1631 PMC2834784

[7] C. Wang , Y. Guan , M. Lv , R. Zhang , Z. Guo , X. Wei , X. Du , J. Yang , T. Li , Y. Wan , X. Su , X. Huang , Z. Jiang , Immunity 2018, 48, 675. 29653696 10.1016/j.immuni.2018.03.017

[8] Z. Zhao , Z. Ma , B. Wang , Y. Guan , X. D. Su , Z. Jiang , Cell Rep. 2020, 32, 108053. 32814054 10.1016/j.celrep.2020.108053

[9] R. Zhang , C. Wang , Y. Guan , X. Wei , M. Sha , M. Yi , M. Jing , M. Lv , W. Guo , J. Xu , Y. Wan , X. M. Jia , Z. Jiang , Cell. Mol. Immunol. 2021, 18, 1222. 33767434 10.1038/s41423-021-00669-w PMC8093200

[10] a) C. Xu , H. E. Dobson , M. Yu , W. Gong , X. Sun , K. S. Park , A. Kennedy , X. Zhou , J. Xu , Y. Xu , A. W. Tai , Y. L. Lei , J. J. Moon , J Control Release 2023, 357, 84; 36948420 10.1016/j.jconrel.2023.03.036 PMC10164691

[11] Q. Ni , F. Zhang , Y. Liu , Z. Wang , G. Yu , B. Liang , G. Niu , T. Su , G. Zhu , G. Lu , L. Zhang , X. Chen , Sci. Adv. 2020, 6, eaaw6071. 32206706 10.1126/sciadv.aaw6071 PMC7080439

[12] J. Zhao , Y. Xu , S. Ma , Y. Wang , Z. Huang , H. Qu , H. Yao , Y. Zhang , G. Wu , L. Huang , W. Song , Z. Tang , X. Chen , Adv. Mater. 2022, 34, 2109254. 10.1002/adma.202109254 34984753

[13] X. Chen , F. Meng , Y. Xu , T. Li , X. Chen , H. Wang , Nat. Commun. 2023, 14, 4584. 37524727 10.1038/s41467-023-40312-y PMC10390568

[14] a) F. Baharom , R. A. Ramirez‐Valdez , K. K. S. Tobin , H. Yamane , C. A. Dutertre , A. Khalilnezhad , G. V. Reynoso , V. L. Coble , G. M. Lynn , M. P. Mule , A. J. Martins , J. P. Finnigan , X. M. Zhang , J. A. Hamerman , N. Bhardwaj , J. S. Tsang , H. D. Hickman , F. Ginhoux , A. S. Ishizuka , R. A. Seder , Nat. Immunol. 2021, 22, 41; 33139915 10.1038/s41590-020-00810-3 PMC7746638

[15] G. M. Lynn , C. Sedlik , F. Baharom , Y. Zhu , R. A. Ramirez‐Valdez , V. L. Coble , K. Tobin , S. R. Nichols , Y. Itzkowitz , N. Zaidi , J. M. Gammon , N. J. Blobel , J. Denizeau , P. de la Rochere , B. J. Francica , B. Decker , M. Maciejewski , J. Cheung , H. Yamane , M. G. Smelkinson , J. R. Francica , R. Laga , J. D. Bernstock , L. W. Seymour , C. G. Drake , C. M. Jewell , O. Lantz , E. Piaggio , A. S. Ishizuka , R. A. Seder , Nat. Biotechnol. 2020, 38, 320. 31932728 10.1038/s41587-019-0390-x PMC7065950

[16] a) S. Liu , Q. Jiang , X. Zhao , R. Zhao , Y. Wang , Y. Wang , J. Liu , Y. Shang , S. Zhao , T. Wu , Y. Zhang , G. Nie , B. Ding , Nat. Mater. 2021, 20, 421; 32895504 10.1038/s41563-020-0793-6

[17] a) M. Abdollahifar , A. R. Karami , N. Haghnazari , C. Karami , Ceram.‐Silik. 2015, 59, 305;

[18] S. K. Wculek , F. J. Cueto , A. M. Mujal , I. Melero , M. F. Krummel , D. Sancho , Nat. Rev. Immunol. 2020, 20, 7. 31467405 10.1038/s41577-019-0210-z

[19] A. Warnatsch , T. D. Tsourouktsoglou , N. Branzk , Q. Wang , S. Reincke , S. Herbst , M. Gutierrez , V. Papayannopoulos , Immunity 2017, 46, 421. 28314592 10.1016/j.immuni.2017.02.013 PMC5965455

[20] L. S. Lin , J. Song , L. Song , K. Ke , Y. Liu , Z. Zhou , Z. Shen , J. Li , Z. Yang , W. Tang , G. Niu , H. H. Yang , X. Chen , Angew. Chem., Int. Ed. 2018, 57, 4902. 10.1002/anie.201712027 29488312

[21] a) J. Chandra , S. M. Teoh , P. Kuo , L. Tolley , A. A. Bashaw , Z. K. Tuong , Y. Liu , Z. Chen , J. W. Wells , C. Yu , I. H. Frazer , M. Yu , J. Immunol. 2021, 206, 987; 33504616 10.4049/jimmunol.2000355

[22] S. Li , M. Luo , Z. Wang , Q. Feng , J. Wilhelm , X. Wang , W. Li , J. Wang , A. Cholka , Y.‐x. Fu , B. D. Sumer , H. Yu , J. Gao , Nat. Biomed. Eng. 2021, 5, 455. 33558734 10.1038/s41551-020-00675-9 PMC8126516

[23] S. G. Reed , M. T. Orr , C. B. Fox , Nat. Med. 2013, 19, 1597. 24309663 10.1038/nm.3409

[24] S. Takizawa , A. Murao , M. Ochani , M. Aziz , P. Wang , J. Leukoc. Biol. 2021, 109, 1019. 33070370 10.1002/JLB.3HI0620-416R PMC8053202

[25] A. Teijeira , M. C. Hunter , E. Russo , S. T. Proulx , T. Frei , G. F. Debes , M. Coles , I. Melero , M. Detmar , A. Rouzaut , C. Halin , Cell Rep. 2017, 18, 857. 28122237 10.1016/j.celrep.2016.12.078

[26] F. Geissmann , M. G. Manz , S. Jung , M. H. Sieweke , M. Merad , K. Ley , Science 2010, 327, 656. 20133564 10.1126/science.1178331 PMC2887389

[27] S. Y. Wu , C. S. Chiang , Cells 2020, 9, 51.

[28] S. C. Eisenbarth , Nat. Rev. Immunol. 2019, 19, 89. 30464294 10.1038/s41577-018-0088-1 PMC7755085

[29] J. A. Villadangos , P. Schnorrer , Nat. Rev. Immunol. 2007, 7, 543. 17589544 10.1038/nri2103

[30] M. L. Kapsenberg , Nat. Rev. Immunol. 2003, 3, 984. 14647480 10.1038/nri1246

[31] L. Ohl , M. Mohaupt , N. Czeloth , G. Hintzen , Z. Kiafard , J. Zwirner , T. Blankenstein , G. Henning , R. Förster , Immunity 2004, 21, 279. 15308107 10.1016/j.immuni.2004.06.014

[32] K. J. Hager , G. Pérez Marc , P. Gobeil , R. S. Diaz , G. Heizer , C. Llapur , A. I. Makarkov , E. Vasconcellos , S. Pillet , F. Riera , P. Saxena , P. Geller Wolff , K. Bhutada , G. Wallace , H. Aazami , C. E. Jones , F. P. Polack , L. Ferrara , J. Atkins , I. Boulay , J. Dhaliwall , N. Charland , M. M. J. Couture , J. Jiang‐Wright , N. Landry , S. Lapointe , A. Lorin , A. Mahmood , L. H. Moulton , E. Pahmer , et al., N. Engl. J. Med. 2022, 386, 2084. 35507508 10.1056/NEJMoa2201300 PMC9127773

[33] R. S. Allan , J. Waithman , S. Bedoui , C. M. Jones , J. A. Villadangos , Y. Zhan , A. M. Lew , K. Shortman , W. R. Heath , F. R. Carbone , Immunity 2006, 25, 153. 16860764 10.1016/j.immuni.2006.04.017

[34] J. Kim , W. A. Li , Y. Choi , S. A. Lewin , C. S. Verbeke , G. Dranoff , D. J. Mooney , Nat. Biotechnol. 2015, 33, 64. 25485616 10.1038/nbt.3071 PMC4318563

[35] a) Y. Li , X. Ma , Y. Yue , K. Zhang , K. Cheng , Q. Feng , N. Ma , J. Liang , T. Zhang , L. Zhang , Z. Chen , X. Wang , L. Ren , X. Zhao , G. Nie , Adv. Mater. 2022, 34, 2109984; 10.1002/adma.202109984 35315546

[36] C. Liu , X. Liu , X. Xiang , X. Pang , S. Chen , Y. Zhang , E. Ren , L. Zhang , X. Liu , P. Lv , X. Wang , W. Luo , N. Xia , X. Chen , G. Liu , Nat. Nanotechnol. 2022, 17, 531. 35410368 10.1038/s41565-022-01098-0

[37] M. Philip , L. Fairchild , L. Sun , E. L. Horste , S. Camara , M. Shakiba , A. C. Scott , A. Viale , P. Lauer , T. Merghoub , M. D. Hellmann , J. D. Wolchok , C. S. Leslie , A. Schietinger , Nature 2017, 545, 452. 28514453 10.1038/nature22367 PMC5693219

[38] R. J. Mitkus , D. B. King , M. A. Hess , R. A. Forshee , M. O. Walderhaug , Vaccine 2011, 29, 9538. 22001122 10.1016/j.vaccine.2011.09.124

[39] a) O. K. Dagher , R. D. Schwab , S. K. Brookens , A. D. Posey , Cell 2023, 186, 1814; 37059073 10.1016/j.cell.2023.02.039

[40] D. B. Johnson , C. A. Nebhan , J. J. Moslehi , J. M. Balko , Nat. Rev. Clin Oncol. 2022, 19, 254. 35082367 10.1038/s41571-022-00600-w PMC8790946

[41] a) T. Song , Y. Xia , Y. Du , M. W. Chen , H. Qing , G. Ma , Adv. Mater. 2021, 33, 2100106; 10.1002/adma.202100106 34013604

[42] D. Zhivaki , F. Borriello , O. A. Chow , B. Doran , I. Fleming , D. J. Theisen , P. Pallis , A. K. Shalek , C. L. Sokol , I. Zanoni , J. C. Kagan , Cell Rep. 2020, 33, 108381. 33207188 10.1016/j.celrep.2020.108381 PMC7727444

[43] M. B. Lutz , N. Kukutsch , A. L. Ogilvie , S. Rössner , F. Koch , N. Romani , G. Schuler , J. Immunol. Methods 1999, 223, 77. 10037236 10.1016/s0022-1759(98)00204-x

