# Supplementary material for: 2D Differential Metallic Immunopotentiators Drive High Diversity and Capability of Antigen‐specific Immunity Against Tumor
Source: Adv Sci (Weinh). 2024 Sep 3;11(40):2405729. doi: 10.1002/advs.202405729 (PMC11516112; doi:10.1002/advs.202405729)
Supplement: Supplementary file 1 — Supporting Information [file ADVS-11-2405729-s001.pdf]

ADVANCED SCIENCE
Open Access

Supporting Information

for *Adv. Sci.*, DOI 10.1002/advs.202405729

2D Differential Metallic Immunopotentiators Drive High Diversity and Capability of Antigen-specific Immunity Against Tumor

Hongze Ren, Anqi Zhu, Wei Yang, Yiwen Jia, Hui Cheng, Ye Wu, Zhengqi Tang, Weifan Ye, Mayu Sun, Yujie Xie, Meihua Yu* and Yu Chen**

Two-Dimensional Differential Metallic Immunopotentiators Drive High Diversity and Capability of Antigen-specific Immunity against Tumor

Hongze Ren, Anqi Zhu, Wei Yang, Yiwen Jia, Hui Cheng, Ye Wu, Zhengqi Tang, Weifan Ye, Mayu Sun, Yujie Xie, Meihua Yu*, Yu Chen**

H. Ren, Y. Jia, H. Cheng, Y. Wu, Z. Tang, W. Ye, Y. Xie, M. Yu, Y. Chen
Materdicine Lab, School of Life Sciences, Shanghai University, Shanghai 200444, China.
E-mail: xieyj@shu.edu.cn (Y. Xie), myu@shu.edu.cn (M. Yu), chenyuedu@shu.edu.cn (Y. Chen)

H. Ren, Y. Wu, Y. Xie, Y. Chen
School of medicine, Shanghai University, Shanghai 200444, China.

A. Zhu
Department of Medical Ultrasound, Shanghai Tenth People's Hospital, School of Medicine, Tongji University, Shanghai, China.

W. Yang
Department of Urology, Xinhua Hospital, School of Medicine, Shanghai Jiaotong University, Shanghai 200092, China.

M. Sun
Laboratory Center, Shanghai Municipal Hospital of Traditional Chinese Medicine, Shanghai University of Traditional Chinese Medicine, Shanghai, China.

Supporting Figures:

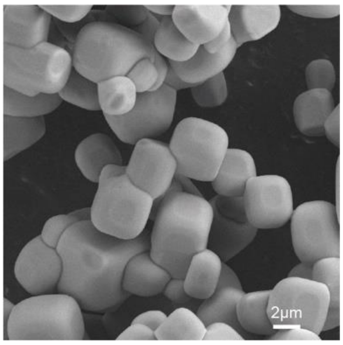

Figure S1. A SEM image of NaCl crystals with smooth surface after recrystallization.

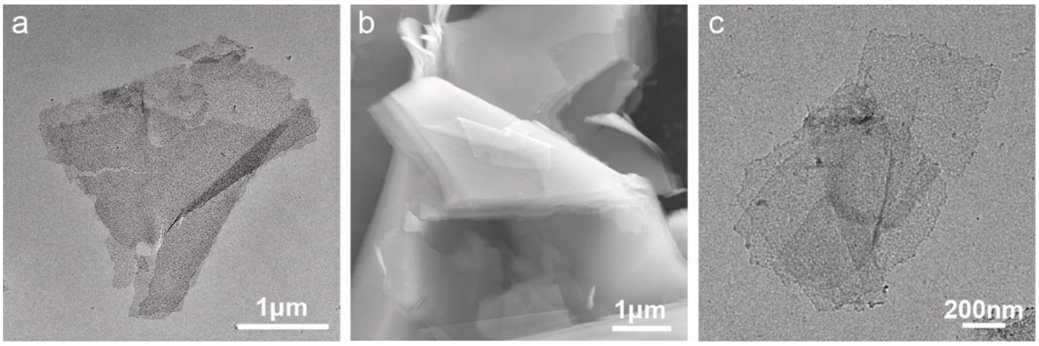

Figure S2. TEM images of ANS before (a) and after (c) ultrasonication, and a SEM image of ANS (b).

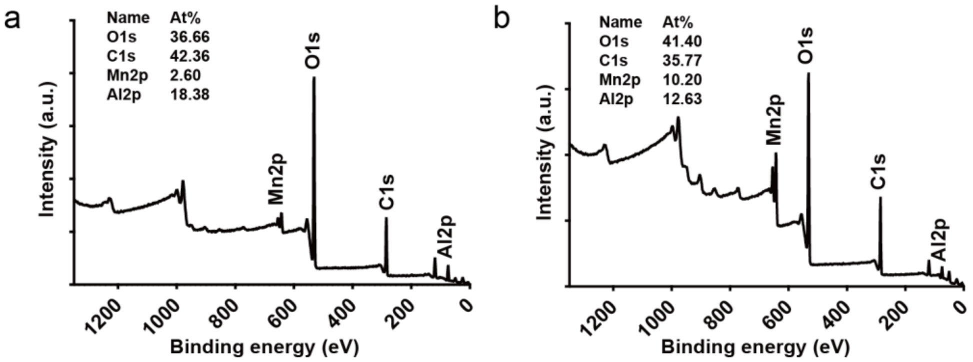

Figure S3. The XPS survey spectra of MANS-L (a) and MANS-H (b).

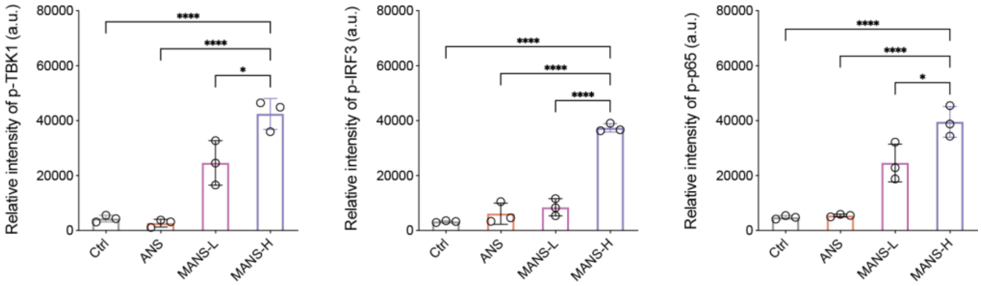

Figure S4. The quantitative analysis of p-TBK1, p-IRF3 and p-p65 of Western blots. Data are presented as means ± SD. P values were determined by one-way ANOVA test. n.s., not significant; *P < 0.05, ****P < 0.0001.

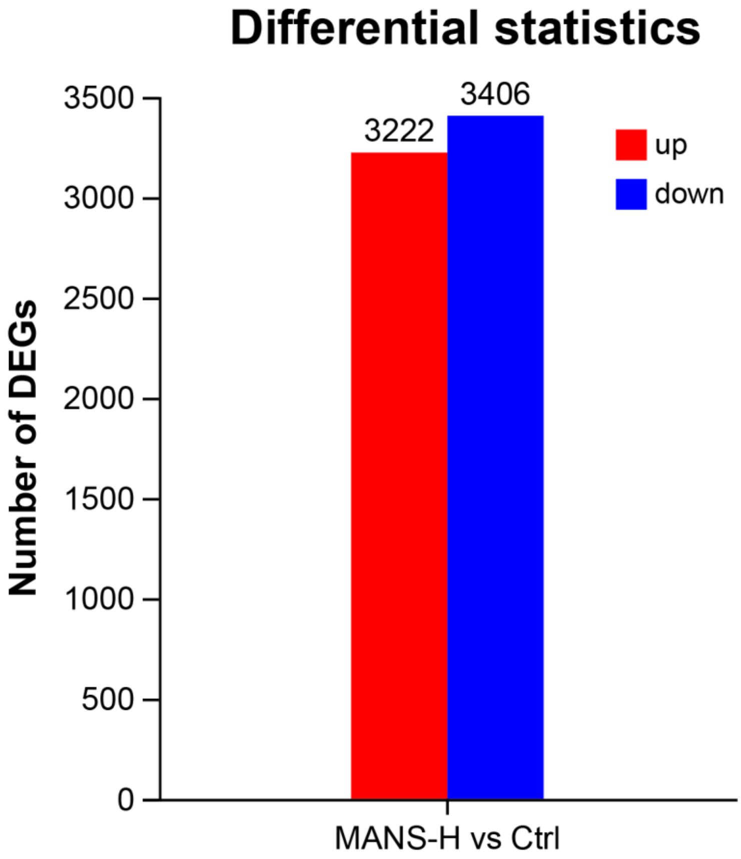

Figure S5. The upregulation and downregulation genes between Ctrl and MANS-H analyzed by bulk RNA sequencing.

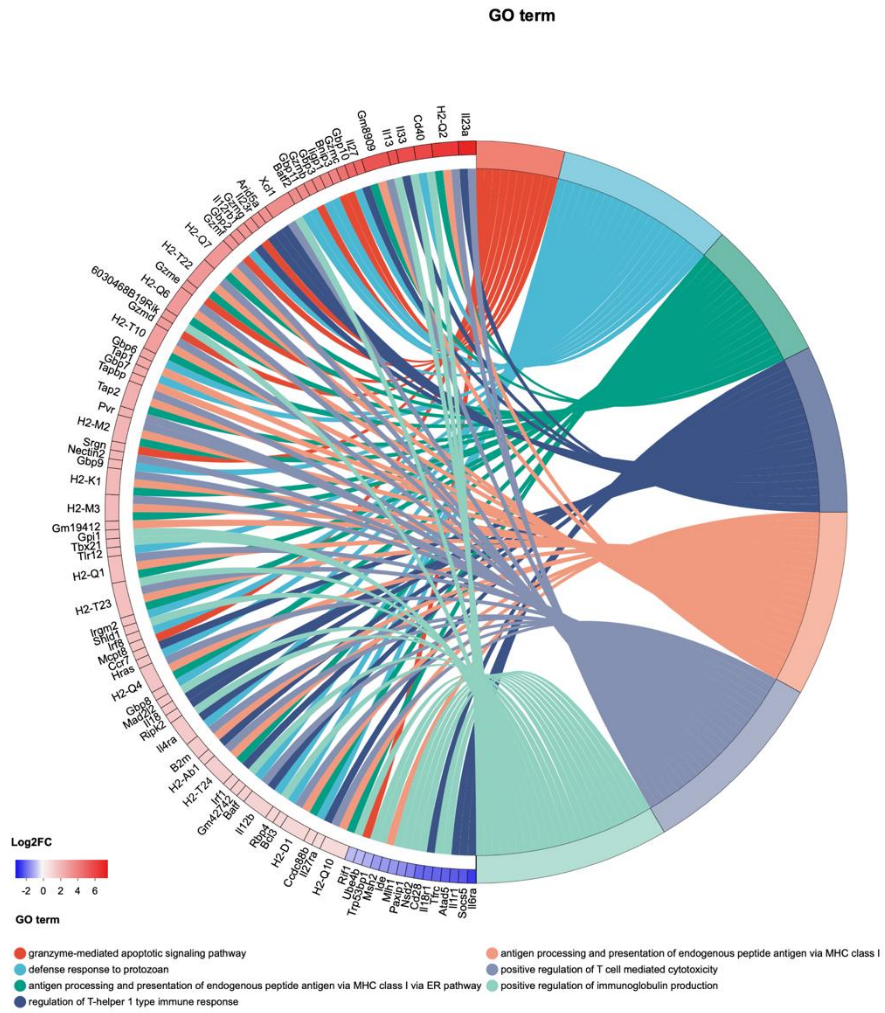

Figure S6. GO biological process terms in the differential genes of the immunological processes.

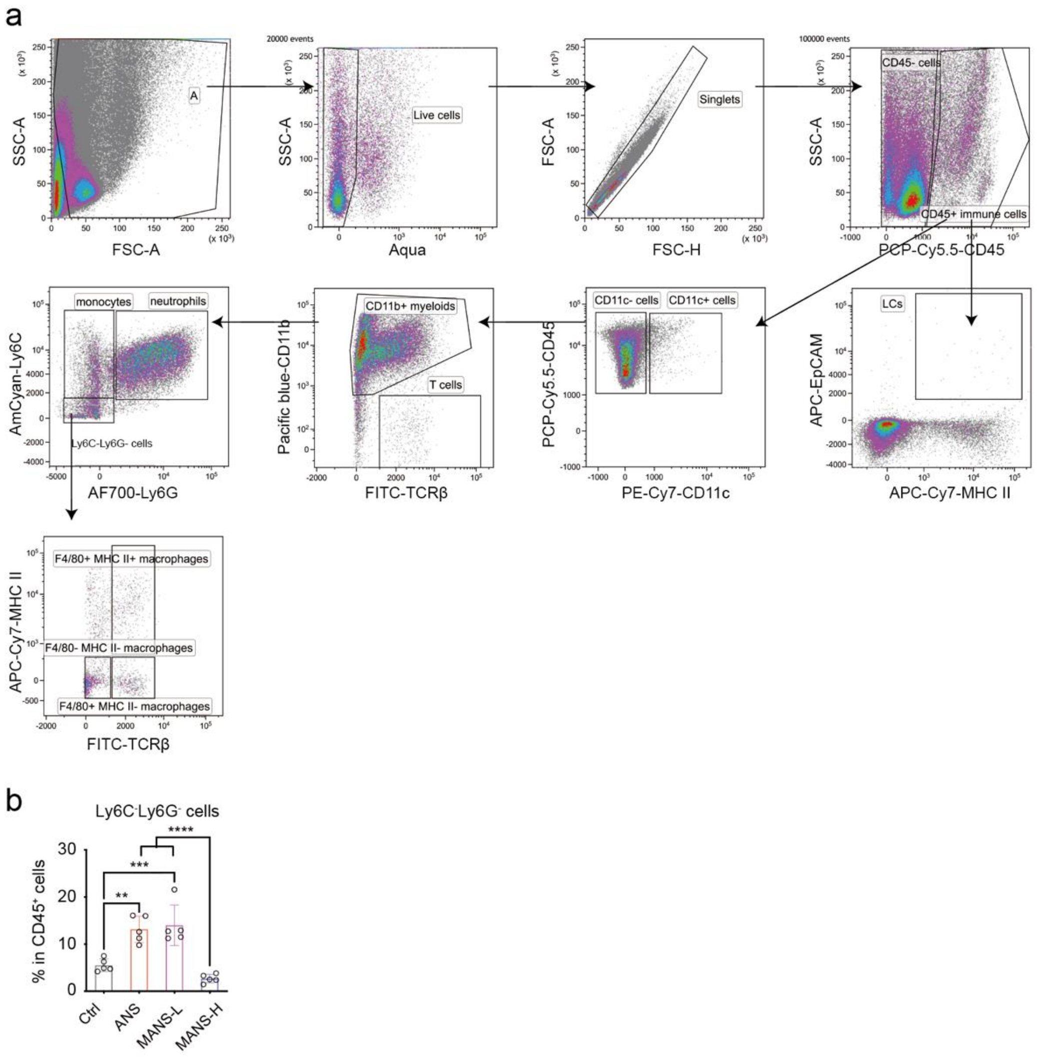

Figure S7. (b) Flow cytometry gating strategy of innate immune cells at the injection site of skins collected from C57BL/6J mice at 48 h post subcutaneous injection with ANS, MANS-L or MANS-H. (b) The percentages of Ly6C$^-$Ly6G$^-$ cells in CD45$^+$ immune cells in the skin at 48 h post immunization of ANS, MANS-L or MANS-H. Data are presented as means ± SD. P values were determined by one-way ANOVA test. **p < 0.01, ***p < 0.001, ****p < 0.0001.

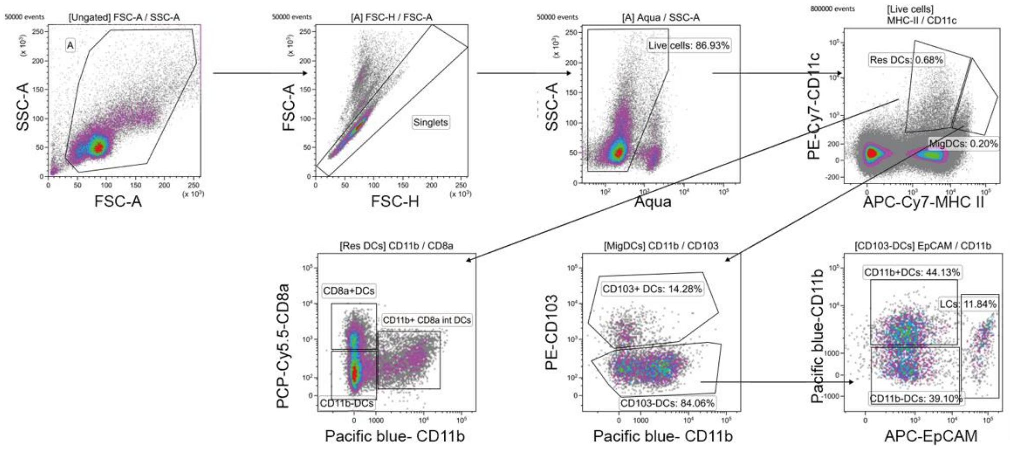

Figure S8. The gating strategy of distinct phenotypes of dendritic cells collected from dLNs after immunization with distinct formulations after 48 h.

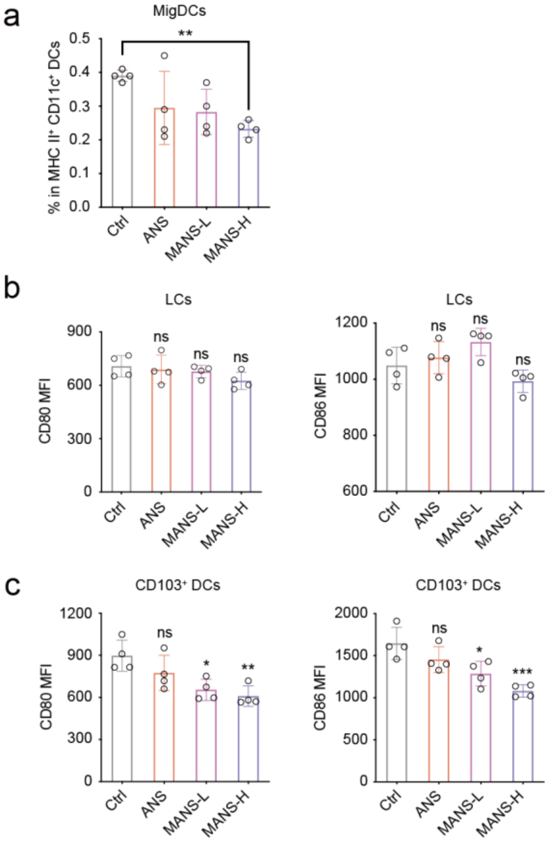

Figure S9. a) Percentage of MigDCs in MHCII$^+$CD11c$^+$ DCs. b) MFI of CD80 and CD86 on LCs in MigDCs. c) MFI of CD80 and CD86 on CD103$^+$ DCs in MigDCs. Data are presented as means ± SD. P values were determined by one-way ANOVA test. n.s., not significant; *P < 0.05, **p < 0.01, ***p < 0.001.

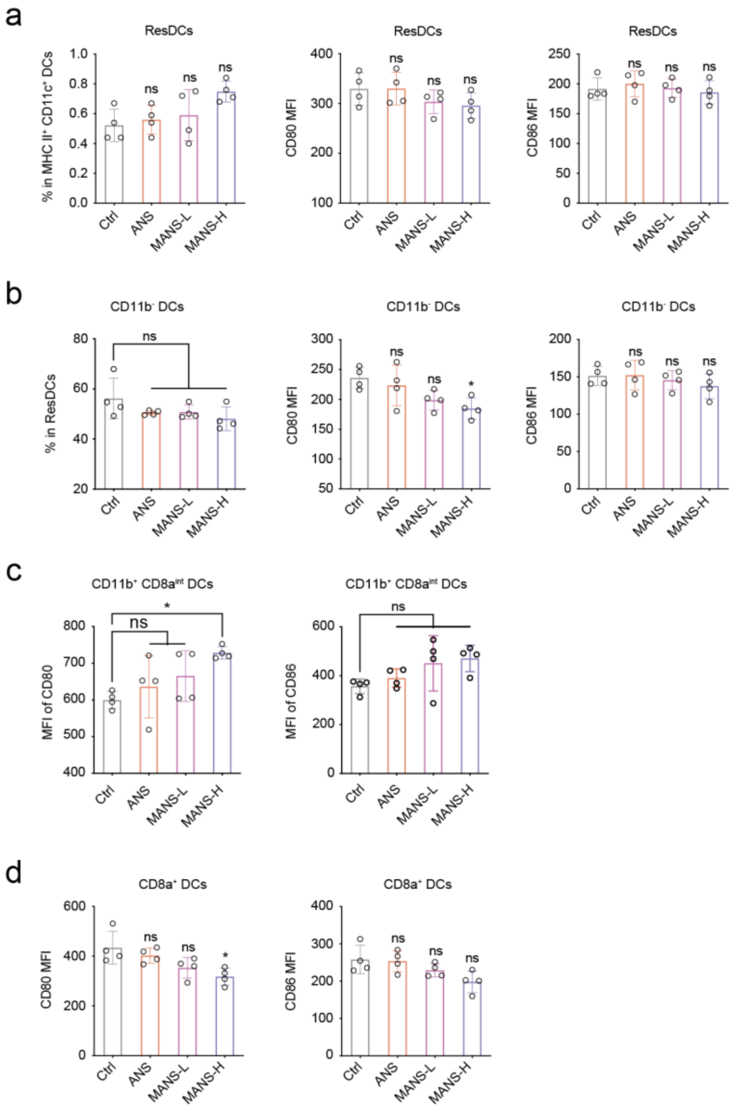

Figure S10. a) The proportion of ResDCs in MHC II^{+}CD11c^{+} DCs and MFI of CD80 and CD86 in ResDCs. b) The proportion of CD11b^{-} DCs in ResDCs and MFI of CD80 and CD86 in CD11b^{-} DCs. c) MFI of CD80 and CD86 in CD11b^{+}CD8a^{int} DCs. d) MFI of CD80 and CD86 in CD8a^{+} DCs. Data are presented as means ± SD. P values were determined by one-way ANOVA test. n.s., not significant, *$P < 0.05$.

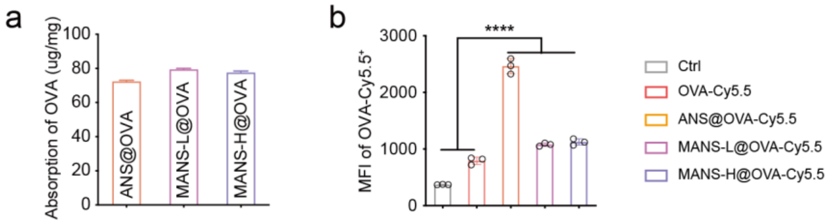

Figure S11. a) The loading capability of OVA of different PAH-NSs. b) MFI of OVA-Cy5.5$^+$ BMDCs co-cultured with different treatments. Data are presented as means ± SD. P values were determined by one-way ANOVA test. ****P < 0.0001.

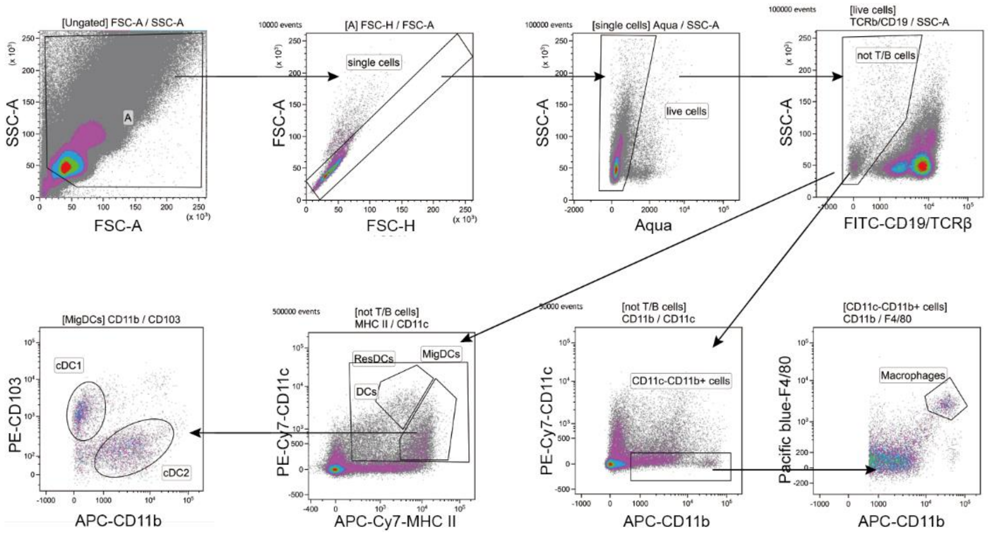

Figure S12. The gating strategy of distinct phenotypes of OVA-Cy5.5^{+} DCs collected from dLNs after immunization with distinct formulations after 24 h, 48 h and 72 h.

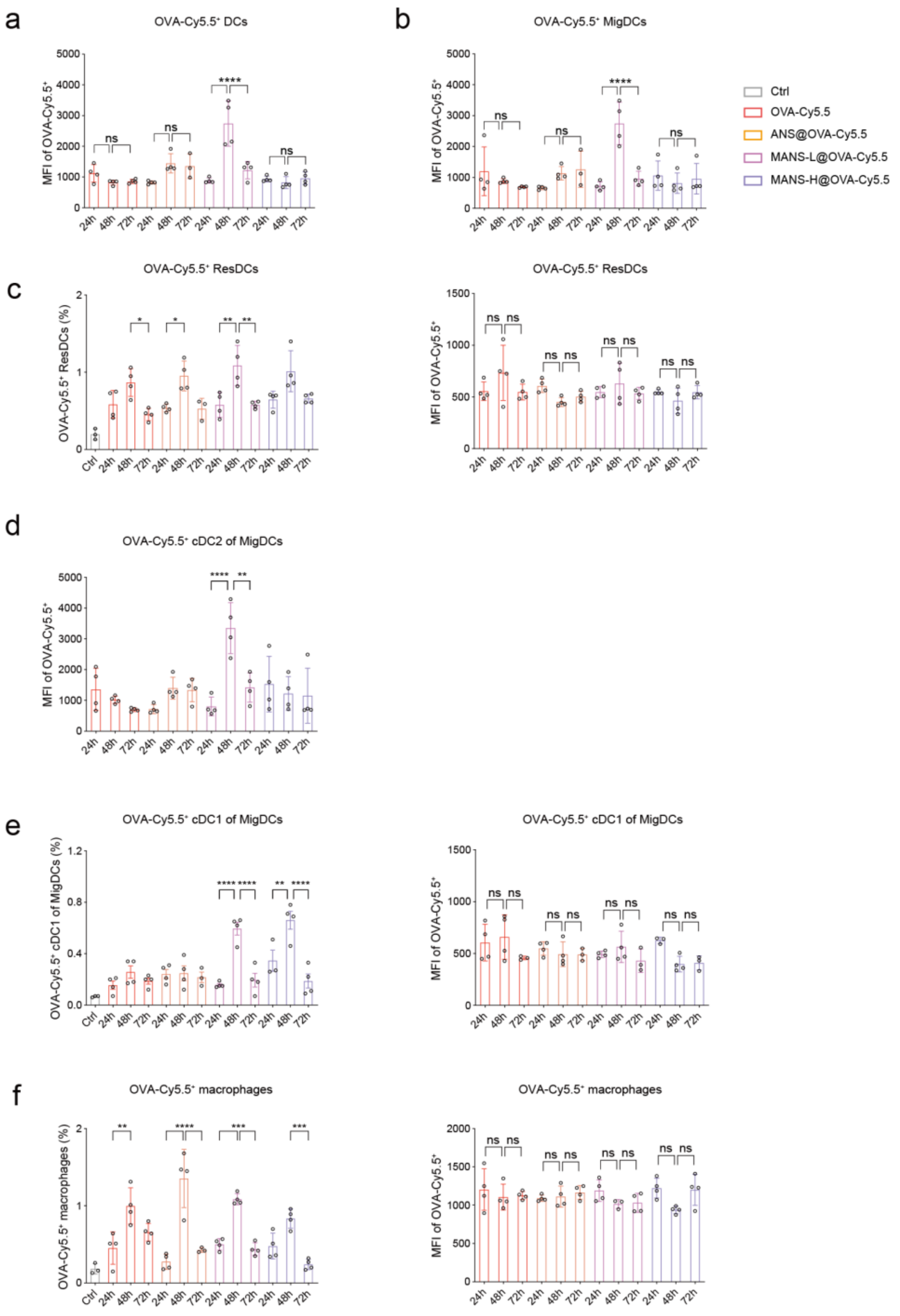

Figure S13. a-b) MFI of OVA-Cy5.5$^+$ DCs (a), OVA-Cy5.5$^+$ MigDCs (b) in dLNs at distinct time points post injection. c) The percentage and MFI of OVA-Cy5.5$^+$ ResDCs in dLNs at distinct time points post injection. d) MFI of OVA-Cy5.5$^+$ cDC2 of MigDCs in dLNs at distinct time points post injection. e-f) The percentage and MFI of OVA-Cy5.5$^+$ cDC1 of MigDCs (e) and OVA-Cy5.5$^+$ macrophages (f) in dLNs at distinct time points post injection. Data are presented as means ± SD. P values were determined by one-way ANOVA test. ns, p>0.05, *p < 0.05, **p < 0.01, ***p < 0.001, ****p < 0.0001.

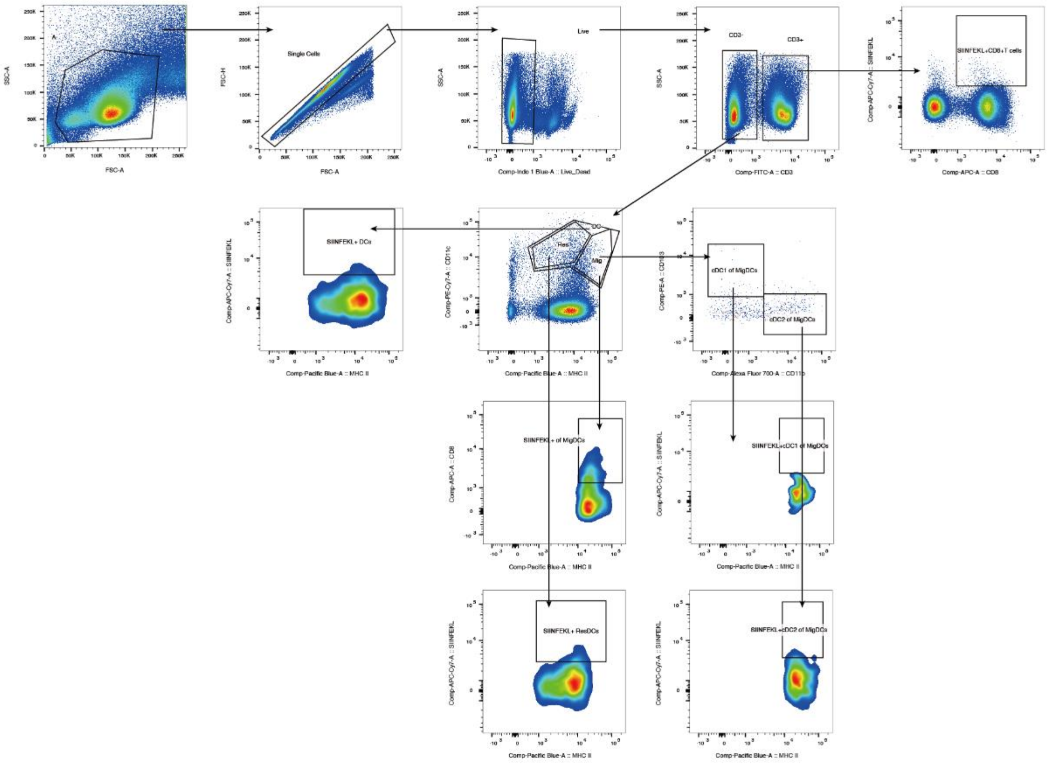

Figure S14. The gating strategy of MHC I-SIINFEKL^{+} DCs, MigDCs, ResDCs, cDC1, cDC2 and CD8^{+} T cells in skin dLNs of C57BL/6J mice immunized MANS-H@OVA.

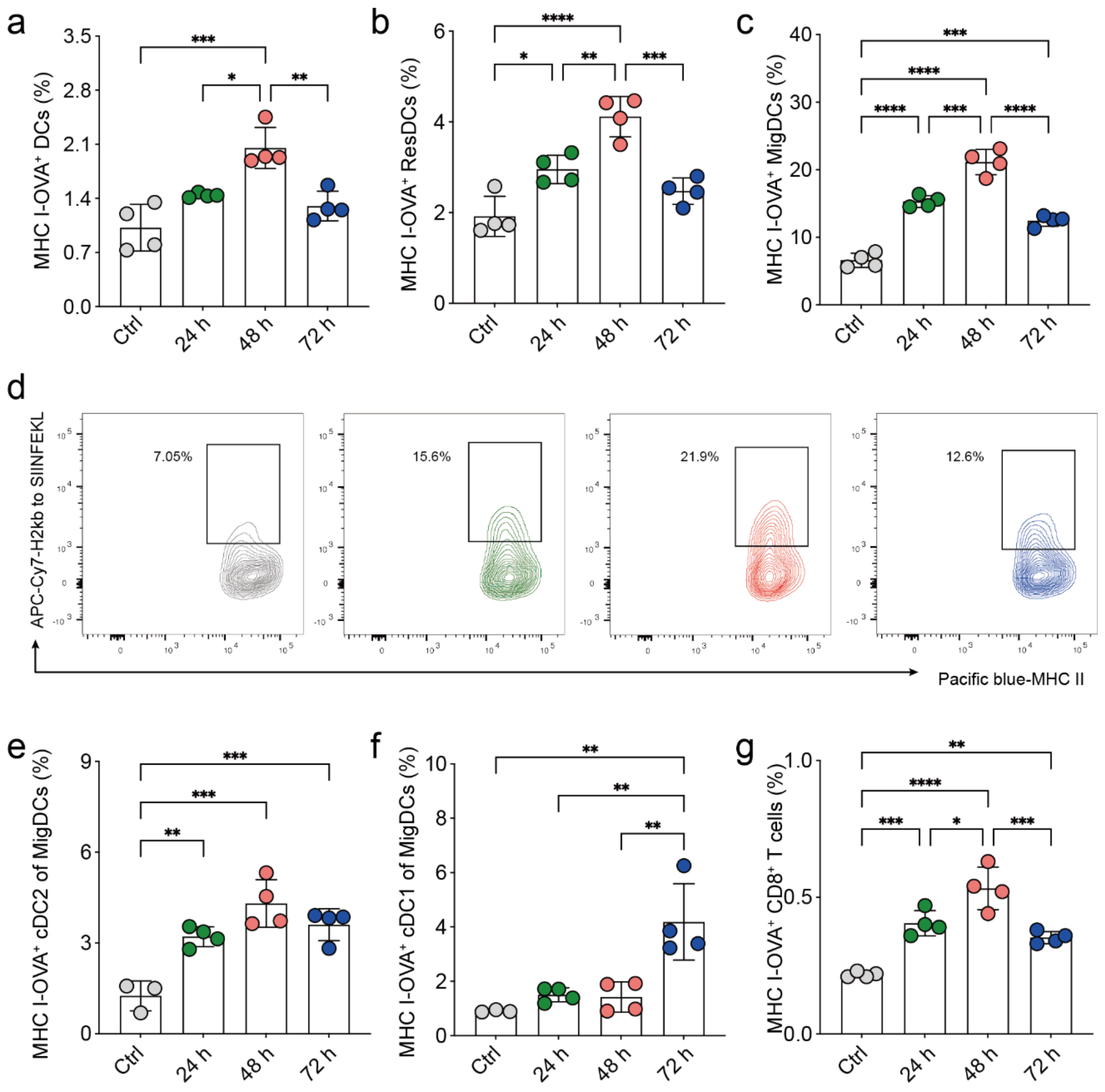

Figure S15. The percentages of MHC I-SIINFEKL expressing DCs (a), ResDCs (b), MigDCs (c), cDC2 of MigDCs (e), cDC1 of MigDCs (f) and CD8^{+} T cells (g), and representative dot plots of MHC I-SIINFEKL expressing MigDCs (d) in dLNs at distinct timepoints post immunizaiton of MANS-H@OVA in C57BL/6J mice. Data are presented as means ± SD. P values were determined by one-way ANOVA test. ns, p>0.05, *p < 0.05, **p < 0.01, ***p < 0.001, ****p < 0.0001.

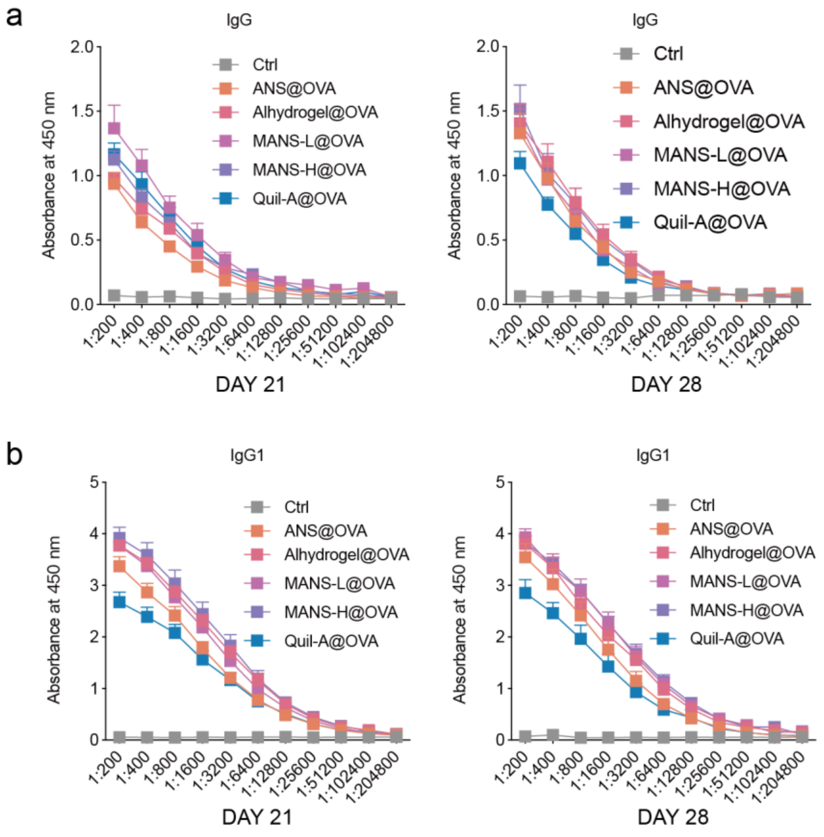

Figure S16. a-b) The absorbance curves at 450 nm for OVA specific IgG antibodies (a) and IgG1 antibodies (b) at different dilutions and timepoints. Data are presented as means ± SEM.

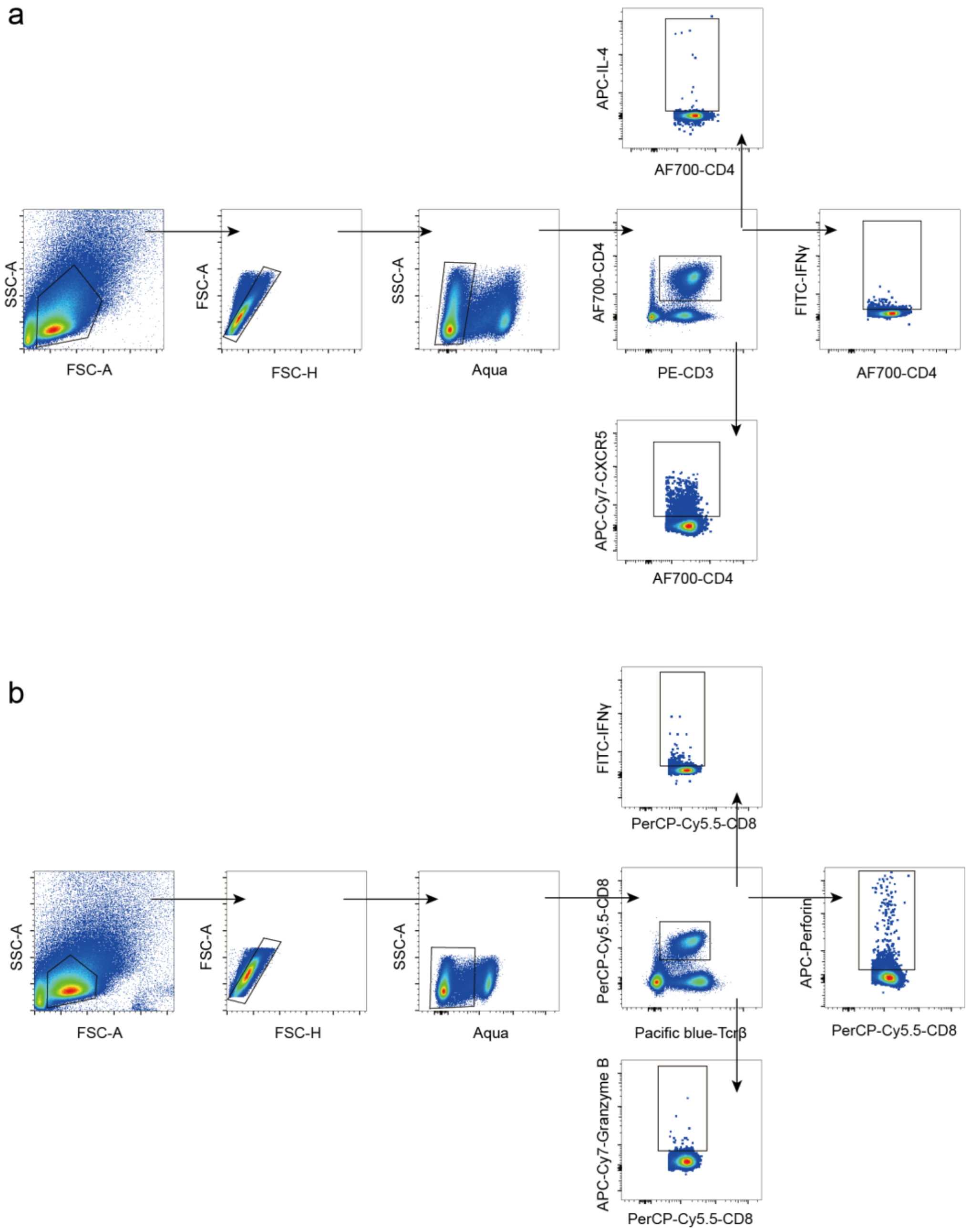

Figure S17. a-b) Gating strategies of OVA specific CD8$^+$ T cells (a) and CD4$^+$ T cells (b) in spleen from C57BL/6J mice immunized with different adjuvanted OVA vaccines.

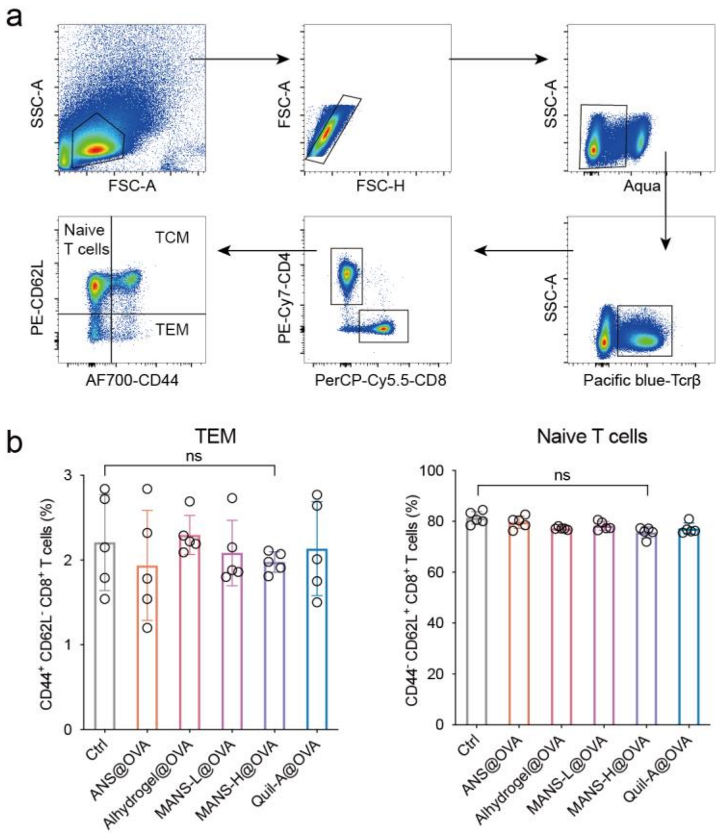

Figure S18 a-b) Gating strategies of distinct phenotypes T memory cells (a) and the fractions of TEM and naïve T cells in CD8$^+$ T cells (b). Data are presented as means ± SD. P values were determined by one-way ANOVA test. ns, $p>0.05$.

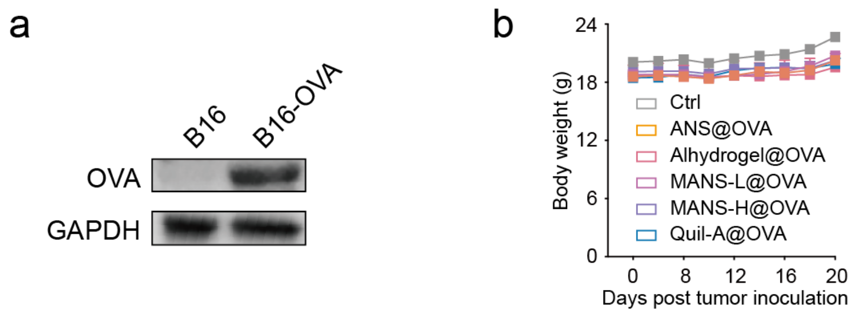

Figure S19. a) The western blot of B16 and B16-OVA cells. b) The body weight curves of mice during the B16-OVA tumor treatment. Data are presented as means ± SEM.

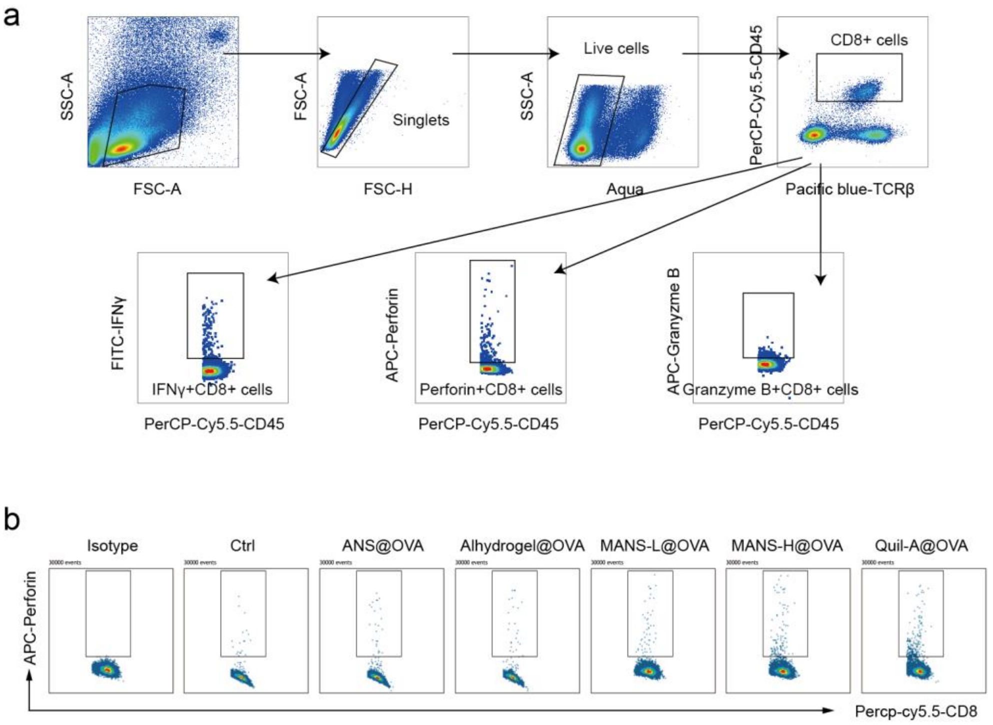

Figure S20. a) The flow cytometry gating strategy for the analysis of IFNγ$^+$CD8$^+$ T cells, Perforin$^+$CD8$^+$ T cells and granzyme B$^+$CD8$^+$ T cells of spleen from C57BL/6J mice bearing B16-OVA tumor after restimulation with OVA257-264 peptide. b) Representative flow cytometry dot plots of Perforin$^+$CD8$^+$ T cells.

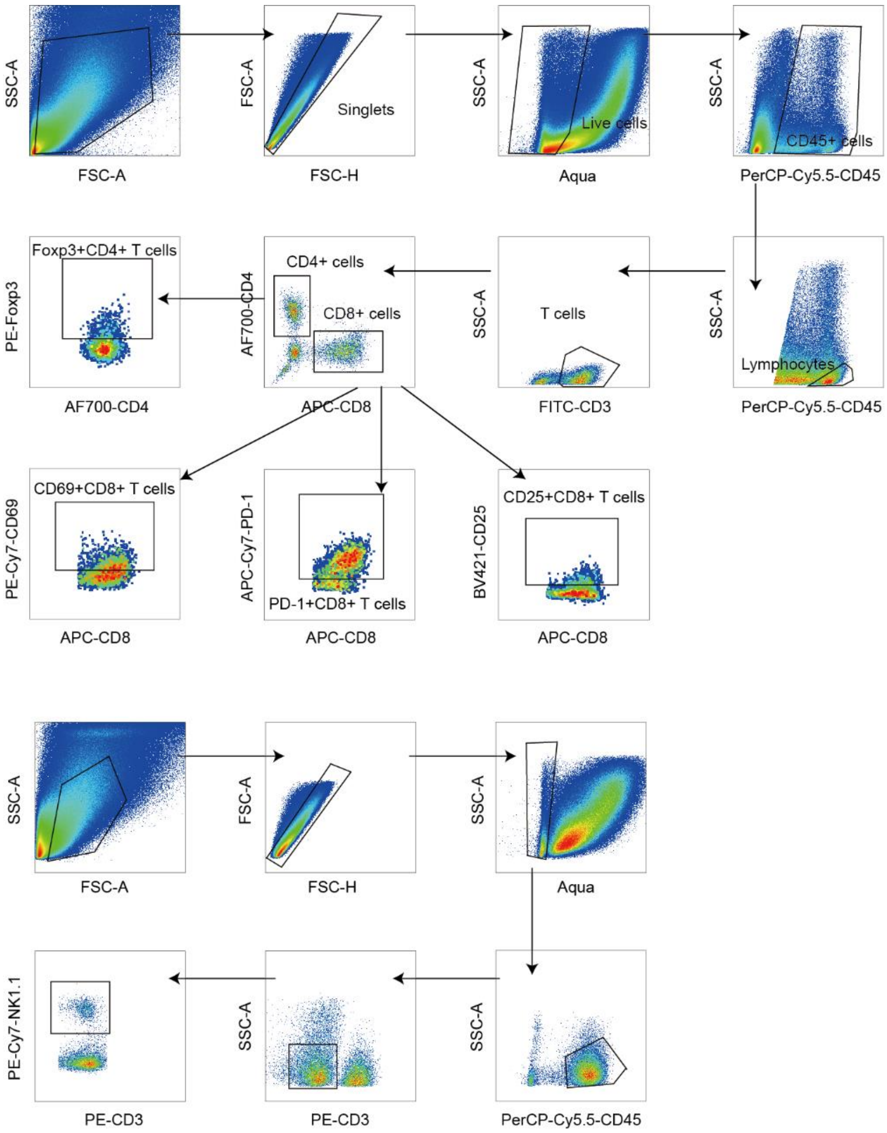

Figure S21. The flow cytometry gating strategy for the analysis infiltrating immune cells in B16-OVA tumor.

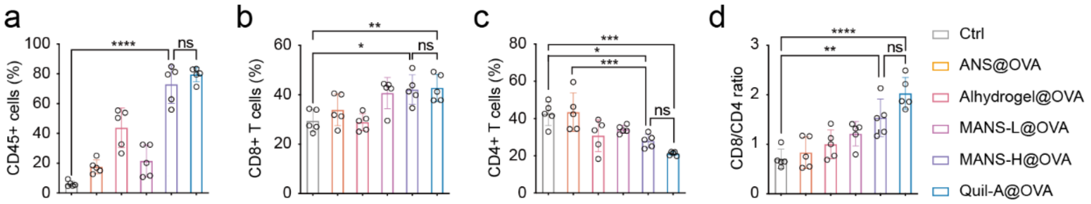

Figure S22 a-d) The fractions of tumor infiltrating $CD45^+$ cells (a), $CD8^+$ T cells (b), $CD4^+$ T cells (c) and CD8/CD4 ratio (d). Data are presented as means ± SD. P values were determined by one-way ANOVA test. ns, p>0.05, *p < 0.05, **p < 0.01, ***p < 0.001, ****p < 0.0001.

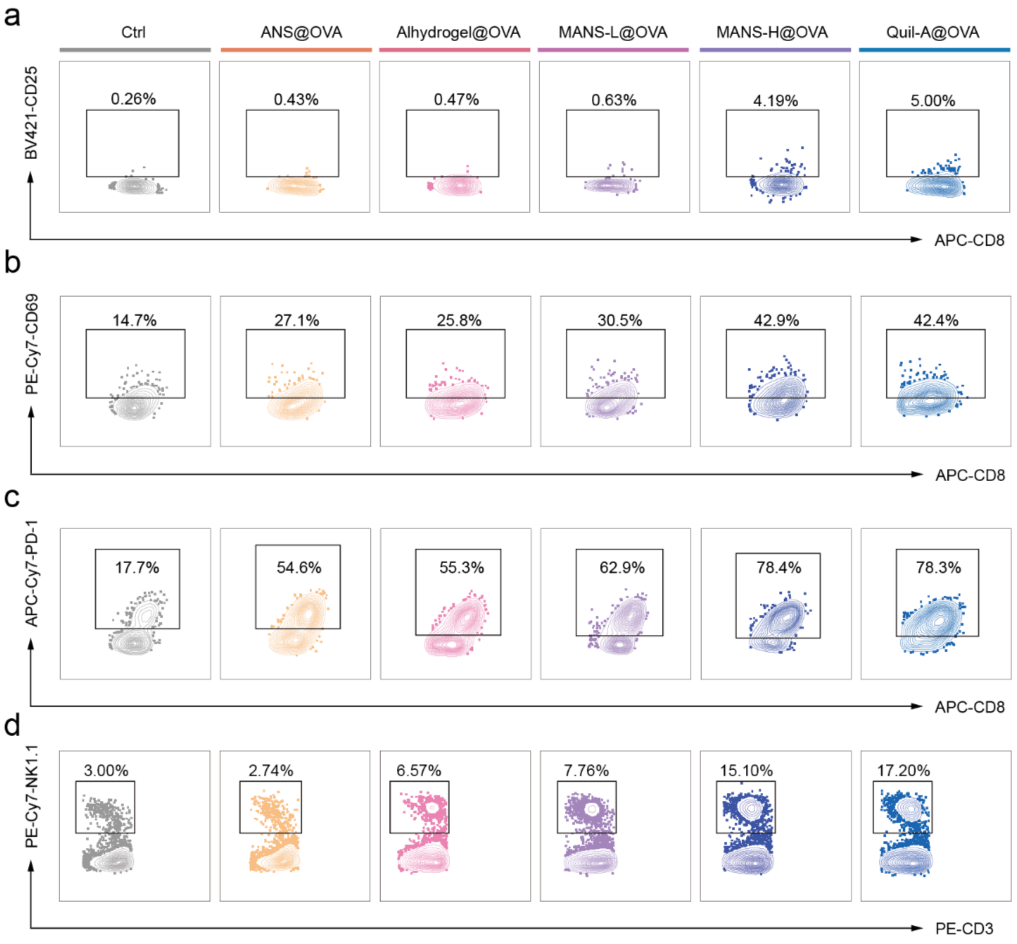

Figure S23. a-d) Representative flow cytometry dot plots of tumor infiltrating $CD25^+CD8^+$ T cells (a), $CD69^+CD8^+$ T cells (b), $PD\text{-}1^+CD8^+$ T cells (c) and NK (d).

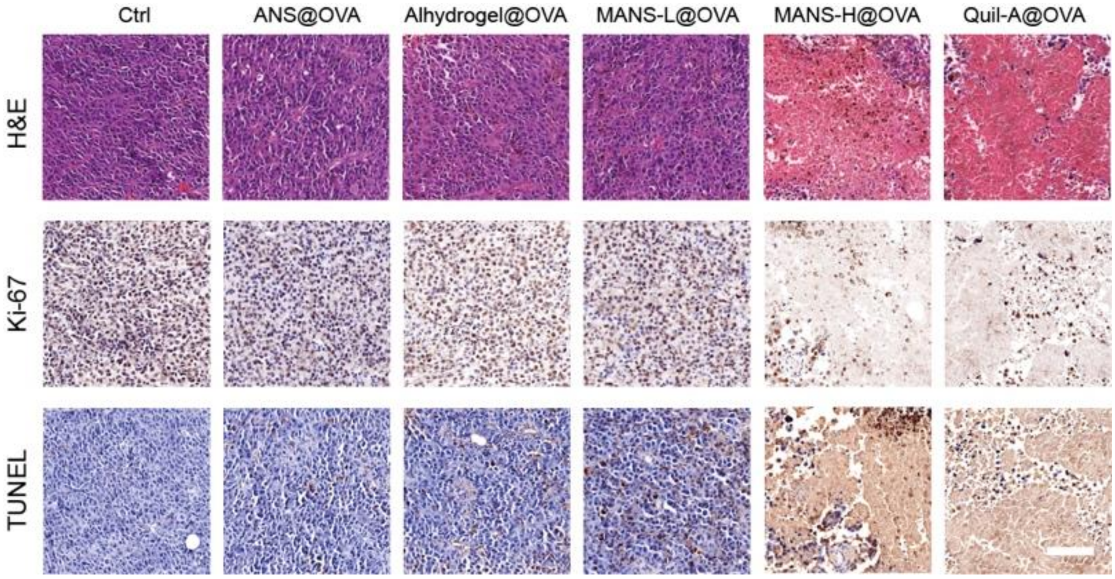

Figure S24. H&E, Ki-67 and TUNEL staining of B16-OVA tumor collected from mice 20 days treated with distinct vaccination strategies.

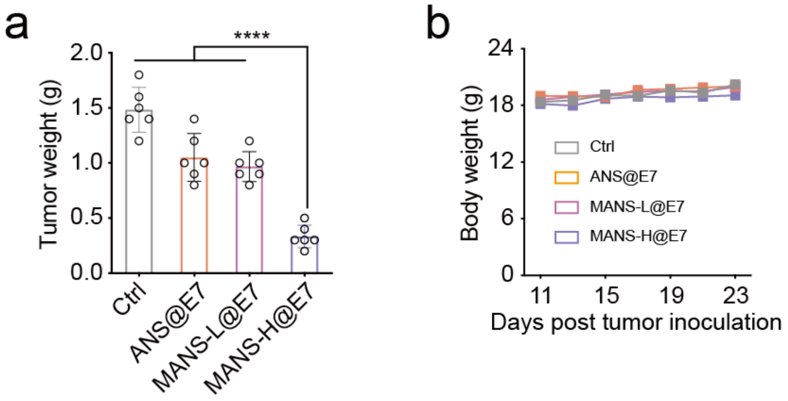

Figure S25. a-b) The TC-1 tumor weight curves (a) and body weight (b) of mice with distinct treatments. Data are presented as means ± SEM. P values were determined by one-way ANOVA test. ****p < 0.0001.

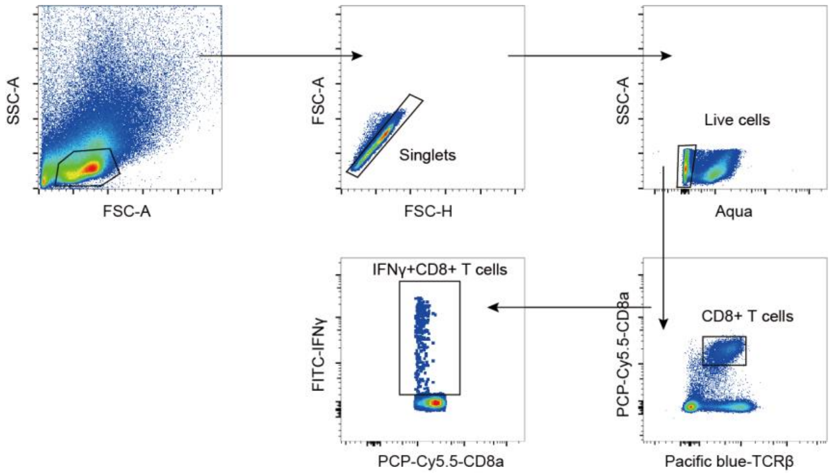

Figure S26. The flow cytometry gating strategy for the analysis of IFN-γ^{+}CD8^{+} T cells of spleen in TC-1 tumor after restimulation with HPV16 E7 peptide (49–57) RAHYNIVTF (GF001).

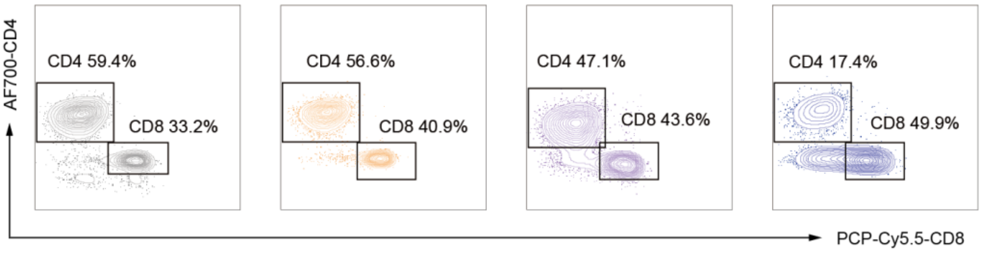

Figure S27. Representative flow cytometry dot plots of CD4$^+$ T cells and CD8$^+$ T cells in TC-1 tumor.

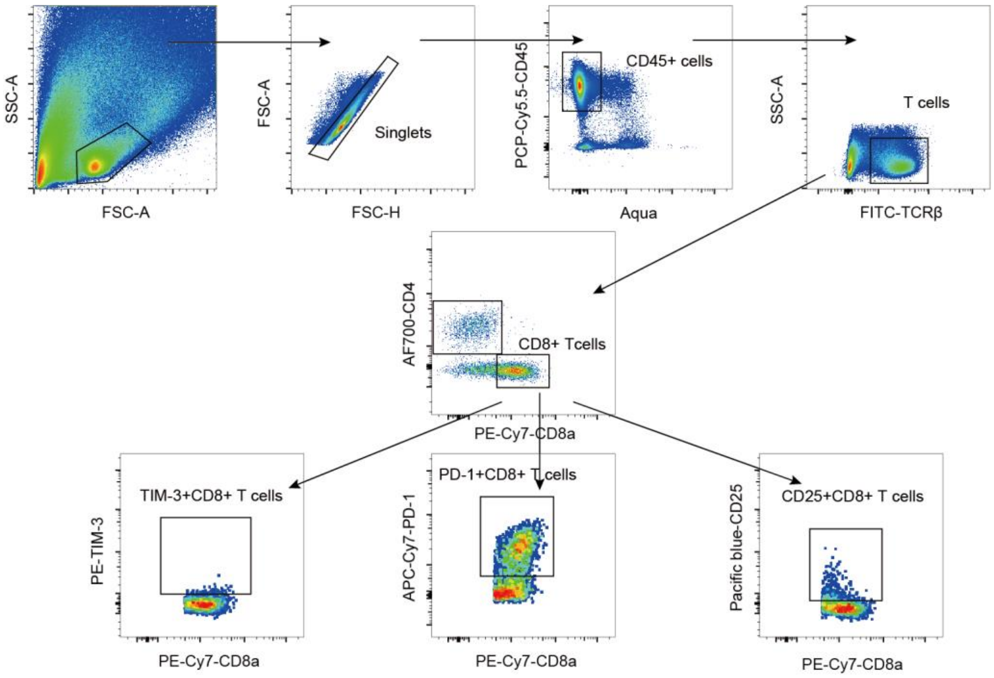

Figure S28. The flow cytometry gating strategy for the analysis infiltrating immune cells in TC-1 tumor.

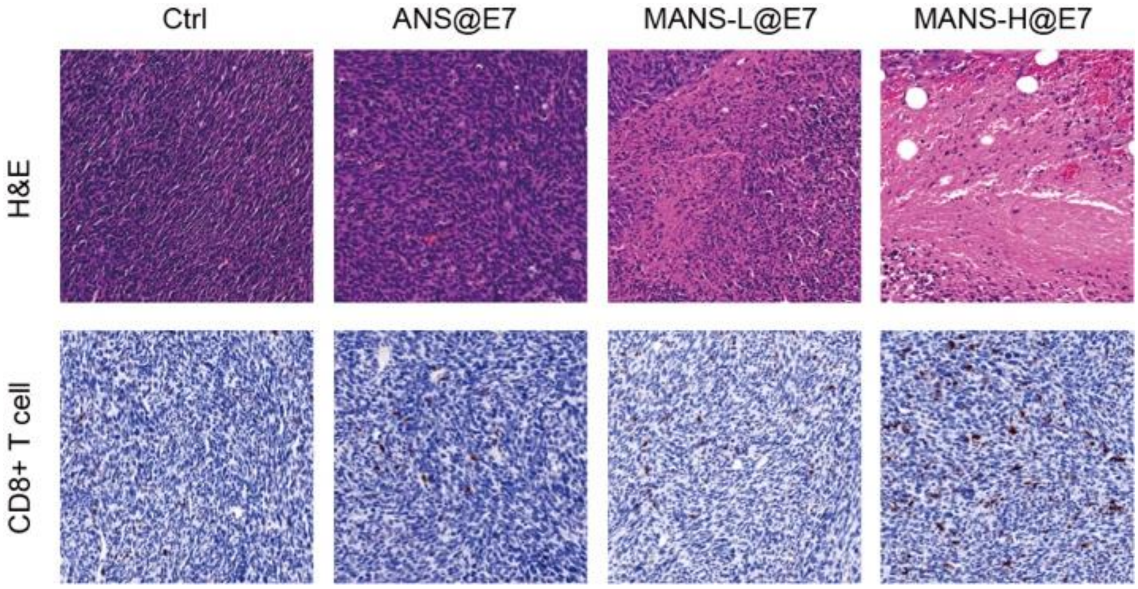

Figure S29. H&E staining and CD8^{+} T cells staining of TC-1 tumor collected from mice 23 days treated with distinct nanovaccine.

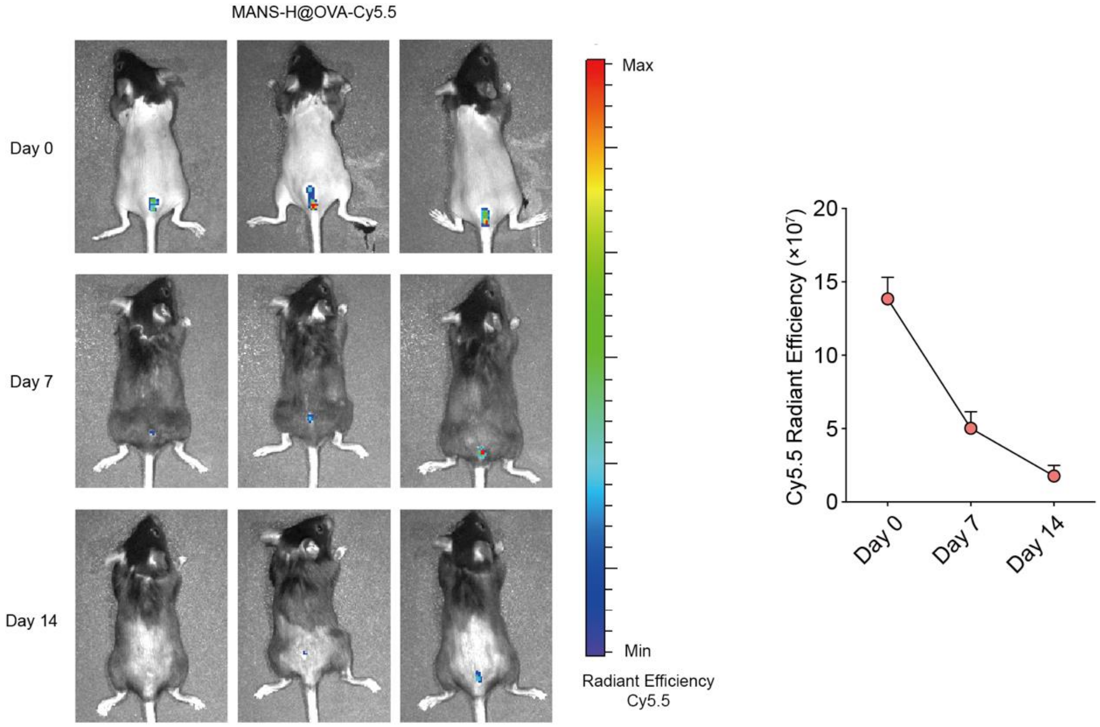

Figure S30. In vivo Cy5.5 fluorescence images at the vaccination location (left) and corresponding total radiant efficiency (right) at different timepoints post s.c. injection of MANS-H@OVA-Cy5.5.

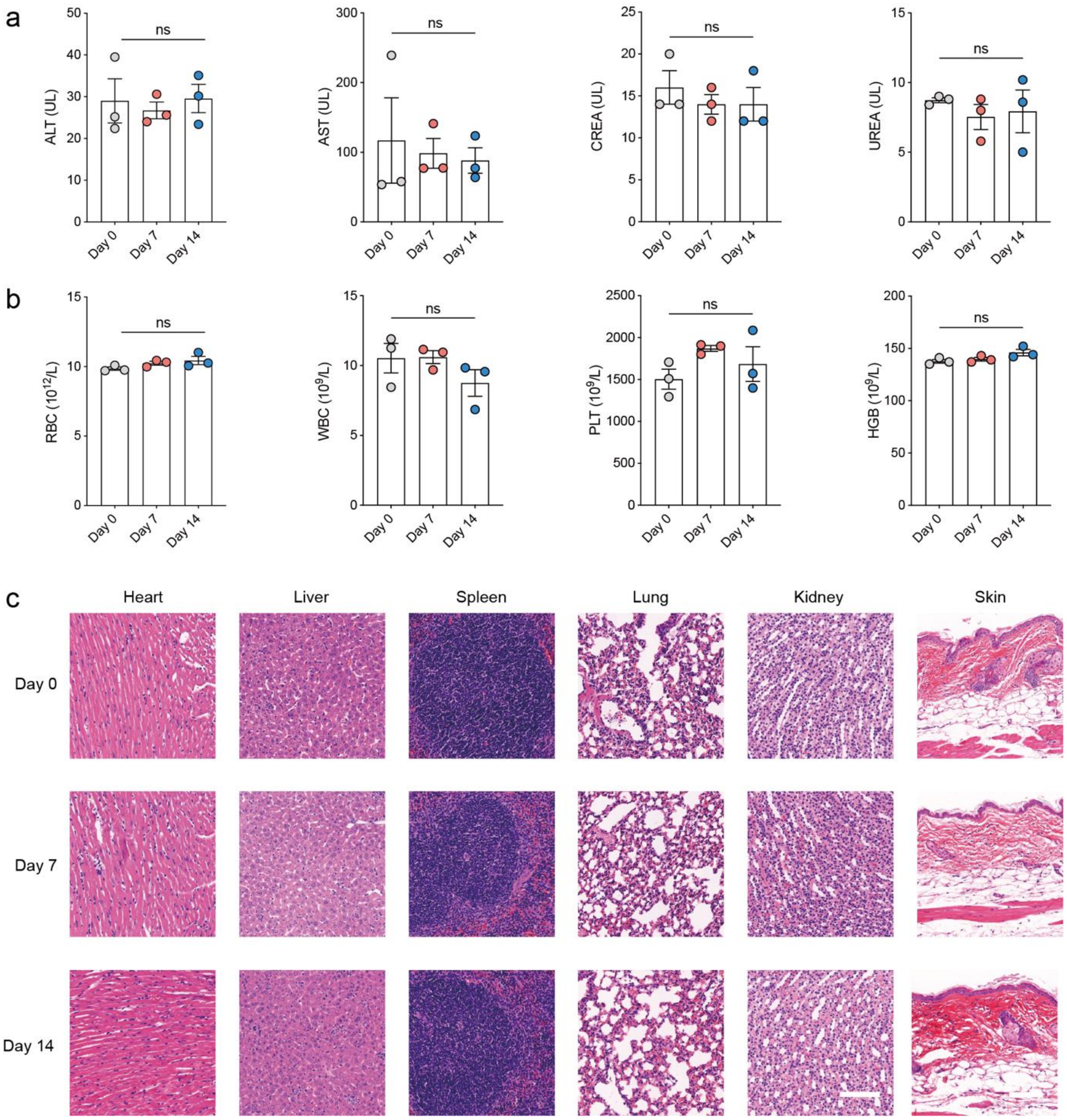

Figure S31. In vivo biosafety evalution of MANS-H. a) serum biochemical analysis, b) blood routine test, and c) H&E staining images of major organs and skin tissues at injection sties from mice post adminisration on Day 0, Day 7 and Day 14. Scar bar: 100 μm. Data are presented as means ± SEM. P values were determined by one-way ANOVA test. ns, p>0.05.

Table S1. Antibody details list.

Antibodies	Fluorophore	Agent	Cat. No.	Clone
Anti-mouse CD16/32	/	Biolegend	101302	93
LIVE/DEAD™ fixable Aqua	/	Invitrogen™	L34966	/
Rat IgG2b, κ	Alexa Flour 647	Biolegend	400626	RTK4530
Anti-mouse Foxp3	Alexa Flour 647	Biolegend	126407	MF-14
Anti-mouse EpCAM	APC	Biolegend	118213	G8.8
Anti-mouse IL-4	APC	Biolegend	504105	11B11
Rat IgG1, κ	APC	Biolegend	400411	RTK2071
Anti-mouse CD11b	APC	Biolegend	101212	M1/70
Anti-mouse Perforin	APC	Biolegend	154403	S16009B
Rat IgG2a, κ	APC	Biolegend	400511	RTK2758
Anti-mouse Ly6G (Gr-1)	Alexa Flour 700	eBioscience	56-5931-80	RB6-8C5
Anti-mouse CD4	Alexa Flour 700	Biolegend	100536	RM4-5
Anti-mouse CD11b	Alexa Flour 700	Biolegend	101222	M1/70
Anti-mouse CD44	Alexa Flour 700	Biolegend	103026	IM7
Anti-mouse CXCR5	APC-Cy7	Biolegend	145525	L138D7
Anti-mouse PD-1	APC-Cy7	Biolegend	135223	29F.1A12
Anti-mouse Granzyme B	APC-Cy7	Biolegend	372227	QA16A02
Mouse IgG1, κ	APC-Cy7	Biolegend	400127	MOPC-21
Anti-mouse MHC II	APC-Cy7	Biolegend	107627	M5/114.15.2
Anti-mouse Ly-6C	Brilliant Violet 510	Biolegend	128033	HK1.4
Anti-mouse CD80	FITC	Biolegend	104705	16-10A1
Anti-mouse F4/80	FITC	eBioscience	11-4801-81	BM8
Anti-mouse CD19	FITC	Biolegend	115505	6D5
Anti-mouse IFN-γ	FITC	Biolegend	505806	XMG1.2
Anti-mouse TCR-β	FITC	Biolegend	109205	H57-597
Rat IgG1, κ	FITC	Biolegend	400405	RTK2071
Anti-mouse CD86	Pacific blue	Biolegend	105022	GL-1
Anti-mouse TCR-β	Pacific blue	Biolegend	109226	H57-597
Anti-mouse F4/80	Pacific blue	Biolegend	123123	BM8
Anti-mouse CD11b	Pacific blue	Biolegend	101223	M1/70
Anti-mouse CD25	Brilliant Violet 421	Biolegend	101923	3C7
Anti-mouse CD62L	PE	Biolegend	104408	MEL-14
Anti-mouse CD8a	PE	eBioscience	12-0081-82	53-6.7
Anti-mouse CD11c	PE	eBioscience	12-0114-81	N418

Anti-mouse TIM-3	PE	Biolegend	134003	B8.2C12
Anti-mouse CD3	PE	Biolegend	100205	17A2
Anti-mouse CD8a	PE-Cy7	Biolegend	162312	S18018E
Anti-mouse CD4	PE-Cy7	Biolegend	100422	GK1.5
Anti-mouse CD11c	PE-Cy7	Biolegend	117318	N418
Anti-mouse CD69	PE-Cy7	Biolegend	104512	H1.2F3
Anti-mouse NK1.1	PE-Cy7	Biolegend	156513	S17016D
Anti-mouse CD8a	Percp-cy5.5	Biolegend	100733	53-6.7
Anti-mouse CD45	Percp-cy5.5	Biolegend	103131	30-F11
